# Heterogeneous Evolution Among SARS‐CoV‐2 Genes and Variants of Concern

**DOI:** 10.1002/jmv.70604

**Published:** 2025-09-12

**Authors:** Luis Daniel González‐Vázquez, Paula Iglesias‐Rivas, David Ferreiro, Miguel Arenas

**Affiliations:** ^1^ CINBIO Universidade de Vigo Vigo Spain; ^2^ Department of Biochemistry, Genetics and Immunology Universidade de Vigo Vigo Spain

**Keywords:** genetic diversity, molecular adaptation, molecular evolution, phylogenetics, rates of evolution, SARS‐CoV‐2 genomic regions, variants of concern

## Abstract

Challenges persist regarding the influence of the severe acute respiratory syndrome coronavirus 2 (SARS‐CoV‐2) on public health, with growing interest in future viral molecular variants. In this context, accurate predictions demand a thorough understanding of the virus's molecular evolution, especially proteins targeted by therapies, where certain discrepancies among studies exist. We analyzed thousands of SARS‐CoV‐2 genomes to assess the rate of evolution and molecular adaptation in the various SARS‐CoV‐2 coding regions. We found an overall low genetic diversity along the genome, with fluctuations over time and among genomic regions, and a notable increase in the Omicron variant, especially in the *S* and *ORF6* genes. We also estimated an overall rate of molecular evolution of approximately 10^−3^ substitutions per site per year, though it varied among genomic regions and over time. Actually, most genomic regions did not follow the strict molecular clock. Regarding selective pressures, the protein‐coding regions of SARS‐CoV‐2 generally exhibited evidence of purifying selection, with local diversifying selection associated with virus transmission and replication. Overall, the molecular evolution of SARS‐CoV‐2 displays heterogeneity among genomic regions and over time. These findings suggest that forecasting SARS‐CoV‐2 molecular evolution is not straightforward and remark the importance of continuing to monitor SARS‐CoV‐2 evolution.

## 1 Introduction

The severe acute respiratory syndrome coronavirus 2 (SARS‐CoV‐2), a Betacoronavirus closely related to the human SARS‐CoV that caused the 2002–2004 SARS outbreak [1], produced the COVID‐19 pandemic with severe social and economic consequences [2]. According to the WHO COVID‐19 dashboard, as of February 2, 2025, more than 777 million confirmed infections and more than 7 million deaths were reported globally. In the last 28 days, more than 75 000 new cases and over 4000 deaths were reported from more than 70 countries. This is particularly remarkable because current reported case numbers likely underestimate the true infection rates because of reduced testing and reporting [3]. Thus, despite vaccination efforts, this virus continues to pose significant challenges to the public health systems, raising ongoing concerns. Currently, attention is understandably focused on what potential future variants may be established and how they could affect therapies [4, 5], making it essential to understand the molecular evolution of the virus.

As SARS‐CoV‐2 spread among humans, it generated multiple molecular variants with distinct transmissibility, disease severity, and immune evasion [4, 6, 7]. In particular, this virus showed the emergence and establishment of several variants of concern (VOCs), namely Alpha, Beta, Gamma, Delta, and Omicron, which rapidly became dominant, outcompeting earlier variants [8]. Recent epidemic waves are driven by Omicron sublineages (e.g., BA.1, BA.2, BA.5), which exhibit enhanced immune escape [8]. As seen in the past, it is expected that future variants derived from current Omicron sublineages could show fitness advantages and relative dominance over previous variants [8]. In general, new variants continue to emerge, and real‐time monitoring of SARS‐CoV‐2 evolution can help in predicting them, providing valuable insights for prevention and treatment. Indeed, it is crucial to understand and consider the molecular mechanisms driving viral diversity and adaptation.

The SARS‐CoV‐2 has an unsegmented genome of approximately 29.9 kb, consisting of single‐stranded positive‐sense RNA [9]. It is encapsulated by a nucleocapsid protein and three additional structural proteins: a membrane protein, an envelope protein, and a spike protein [10]. The genome also contains regions that encode several accessory and nonstructural proteins involved in viral replication [6, 9, 11]. The accumulation of genetic variation in SARS‐CoV‐2 populations promotes the emergence of selected phenotypic traits that can affect key viral mechanisms such as transmission, immune evasion, and pathogenicity [6, 9, 11]. Understanding how genetic diversity varies over time, both across the genome and within specific regions, is essential for characterizing the molecular evolution of SARS‐CoV‐2 and its impact on phenotypic traits. Previous studies showed that during the initial spread of SARS‐CoV‐2 in humans, the genome generally exhibited a moderate rate of evolution, ranging from 10^−3^ to 10^−4^ substitutions per site and year [12]. However, discrepancies exist regarding whether this rate of evolution has varied over time. While some studies suggested significant evolutionary accelerations [13], others proposed only minor deviations from a constant rate of evolution, fitting with the strict molecular clock model [14]. Those studies used relatively small sample sizes (i.e., 122 and 180 genomes in [12] and [13], respectively) and did not compare the rate of evolution among SARS‐CoV‐2 genes. Therefore, additional studies with larger sample sizes and covering all SARS‐CoV‐2 genomic regions are needed to properly evaluate the rate of evolution of this virus. Molecular adaptation is another crucial evolutionary process to consider, as it drives the evolutionary trajectories of each molecular variant. Previous studies showed that molecular adaptation varies among SARS‐CoV‐2 genes; however, significant disagreements exist among these studies (see [6] for a review). For instance, some studies identified purifying selection [15], while others found diversifying selection [16] in the gene coding for the nucleocapsid protein (N gene). We believe that updating knowledge of the molecular evolution of SARS‐CoV‐2 through the comprehensive analysis of large data for each protein‐coding region is essential to properly understanding the complex evolution of this virus. Therefore, we present a thorough analysis of the molecular evolution of SARS‐CoV‐2 at both the genome and protein‐coding region levels, based on the examination of 4500 whole genome sequences.

## 2 Materials and Methods

### 2.1 Data Collection

We constructed two types of genome and coding regions data for different evaluations. Data designed to investigate the evolution from one VOC to another, and data designed to investigate the evolution among sets of variants existing at different time points (Table 1). Indeed, the NCBI identifier, collection date, and country of origin for all sequences used in the study, which also passed filters of quality required by PANGO, are presented in Supporting Information S2: Table S2.

○ The VOCs data included data sets with 250 random genome sequences for each VOC (Alpha, Beta, Gamma, Delta, and Omicron) collected from the NCBI nucleotide database in April 2024. Additionally, we included a data set comprising 250 genome sequences, with 50 sequences from each VOC (hereafter referred to as mixture data). The assignment of sequences to VOCs was performed using the framework *Pangolin* [17]. In addition, we included a data set with 250 sequences for each studied genomic region (details below) for each VOC and mixture of VOCs.
○ The temporal data included data sets of 250 random genome sequences sampled at each 4 months from January 2020 to April 2024, collected from the NCBI nucleotide database, regardless of their variant classification. For each temporal period and studied genomic region (details below), we obtained a data set consisting of 250 sequences. Additionally, we investigated data sets for each studied genomic region containing the sequences sampled across all the studied periods of time (3250 sequences).

**Table 1:** Scheme of the study data.

Type of data	VOC or temporal period	Contents	Number of sequences
VOCs data	Alpha	Whole genome sequences	250
		Coding region sequences (25 region‐specific data)	250
	Beta	Whole genome sequences	250
		Coding region sequences (25 region‐specific data)	250
	Gamma	Whole genome sequences	250
		Coding region sequences (25 region‐specific data)	250
	Delta	Whole genome sequences	250
		Coding region sequences (25 region‐specific data)	250
	Omicron	Whole genome sequences	250
		Coding region sequences (25 region‐specific data)	250
	Mixture (all VOCs)	Whole genome sequences	250
		Coding region sequences (25 region‐specific data)	250
Temporal periods data	January 2020–April 2020	Coding region sequences (25 region‐specific data)	250
	May 2020–August 2020	Coding region sequences (25 region‐specific data)	250
	September 2020–December 2020	Coding region sequences (25 region‐specific data)	250
	January 2021–April 2021	Coding region sequences (25 region‐specific data)	250
	May 2021–August 2021	Coding region sequences (25 region‐specific data)	250
	September 2021–December 2021	Coding region sequences (25 region‐specific data)	250
	January 2022–April 2022	Coding region sequences (25 region‐specific data)	250
	May 2022–August 2022	Coding region sequences (25 region‐specific data)	250
	September 2022–December 2022	Coding region sequences (25 region‐specific data)	250
	January 2023–April 2023	Coding region sequences (25 region‐specific data)	250
	May 2023–August 2023	Coding region sequences (25 region‐specific data)	250
	September 2023–December 2023	Coding region sequences (25 region‐specific data)	250
	January 2024–April 2024	Coding region sequences (25 region‐specific data)	250
	All periods (January 2020–April 2024)	Coding region sequences (25 region‐specific data)	3250

*Note:* We studied two types of data. First, the “VOCs data” (above) included, for each VOC, a data set with 250 whole genome sequences and a data set for each genomic region (details below) containing 250 sequences per region. Additionally, a “mixture data” comprising 250 sequences (50 from each VOC), including a data set of whole genome sequences and a data set for each genomic region. Second, the “temporal periods data” (below) included, for each studied time period, 250 sequences of each genomic region (details below). Additionally, a data set for each genomic region with all the sequences sampled during the studied periods of time, totaling 3250 sequences. The 25 genomic regions studied include the structural genes *E, M, N, S*; the ORFs (*ORF3a, ORF6, ORF7a, ORF7b, ORF8*, and *ORF10*); and the regions of *ORF1ab* that encode for the nonstructural proteins (nsp1, nsp2, nsp3, nsp4, nsp5A, nsp6, nsp7, nsp8, nsp9, nsp10, RdRP, nsp13, nsp14, nsp15 and nsp16).

Sample sizes of 250 sequences per VOC and time point provided statistically robust estimates (see Section 3), as also shown in previous studies [13, 18], while ensuring computational requirements remained feasible. Next, the genome sequences were partitioned into 25 genes and nonstructural protein‐coding regions present in the ORF1ab (excluding nsp11 due to its small size and overlap with RdRP). Specifically, the studied genomic regions included the structural genes E (Envelope), M (Membrane), N (Nucleocapsid), S (Spike), and Open Reading Frames (ORF3a, ORF6, ORF7a, ORF7b, ORF8, ORF10), as well as those encoding for nonstructural proteins in ORF1ab (nsp1, nsp2, nsp3, nsp4, nsp5A, nsp6, nsp7, nsp8, nsp9, nsp10, RdRP, nsp13, nsp14, nsp15, nsp16). These partitions were obtained by individually aligning all the studied genome sequences with the traditional reference genome sequence from Wuhan (GenBank code: NC_045512.2) using *MAFFT* [19] and splitting them according to the NCBI annotation. Next, we added the reference sequence for the corresponding region of interest, and we aligned all the sequences with *MAFFT*. Finally, we adjusted the reading frame according to the reference sequence, which was then removed.

### 2.2 Essential Evolutionary Assessments

We calculated the nucleotide diversity (π) and identified the nucleotide substitution model that best fits each data set using *ModelTest‐NG* [20] under the Bayesian Information Criterion (BIC) [21]. Next, we investigated the potential presence of recombination in each data set using the *GARD* algorithm [22] implemented in *HyPhy* [23]. We found no evidence of recombination in any of the data sets, ensuring that subsequent phylogenetic inferences were not biased by recombination [24, 25, 26]. To further verify the VOC assignment of the sequences of the mixture data through phylogenetic relationships, we used this data to reconstruct a maximum likelihood (ML) phylogenetic tree with *RAxML‐NG* [27], based on the corresponding best‐fitting substitution model of DNA evolution. This analysis confirmed proper assignment (Supporting Information S1: Figure S1).

### 2.3 Analyses of the Rate of Evolution and Molecular Clock

We examined whether the molecular evolution of SARS‐CoV‐2, at both the genome and gene levels, follows a strict molecular clock. For each data set, we inferred an ML phylogenetic tree under the corresponding best‐fitting substitution model with *RAxML‐NG* and performed an ML‐based molecular clock test using *MEGA X* [28]. The results from these analyses indicated that most of the coding regions did not follow the strict molecular clock (see Results for details). Therefore, we applied the relaxed uncorrelated lognormal clock model for subsequent analyses, which was also consistent with previous studies [29, 30]. We estimated the rate of evolution with *Beast 2.6.7* [31]. Given the rapid and broad population expansion of SARS‐CoV‐2 during the pandemic [32], we considered exponential growth of the population size, as also suggested by [12], under an appropriate nucleotide substitution model. Indeed, we calibrated the evolutionary time in *Beast* using the sampling dates of the sequences. We considered the number of MCMC iterations to reach convergence for all parameters (effective sample size [ESS] ≥ 200), as recommended by the developers, and this was also tested using *Bali‐Phy3* [33]. Analyses where convergence was not achieved after a large number of iterations were discarded, with a threshold of 125 M iterations set for exclusion.

### 2.4 Analyses of Molecular Adaptation in Genes and Codon Sites

We studied molecular adaptation through the traditional nonsynonymous synonymous substitution rate ratio *dN/dS*, where purifying (negative selection) corresponds to a ratio below 1, diversifying (positive) selection corresponds to a ratio above 1, and neutral evolution (absence of selection) corresponds to a ratio around 1 [34, 35, 36]. We investigated selection in protein‐coding regions at both the global (entire region) and local (codon site) levels using *dN/dS* estimates from an ML method, which requires a phylogenetic tree reconstructed from the study data. For each coding region, we first reconstructed the ML phylogenetic tree under the corresponding best‐fitting substitution model using *RAxML‐NG*, and then estimated *dN/dS* with the single likelihood ancestor counting (*SLAC*) method [18] implemented in *HyPhy* [23]. This method provides *dN/dS* estimates with a 95% confidence interval. Additionally, we identified positively and negatively selected sites (PSSs and NSSs, respectively), considering significance with a *p*‐value < 0.05, as recommended by the developers of the estimation method [18, 23].

## 3 Results

We present the evolutionary estimates from the whole genome and genomic regions for each VOC and mixed VOCs data, followed by those for the temporal periods data.

### 3.1 Heterogeneous Genetic Diversity, Generally Low but Increasing in Omicron

**Figure 1:**
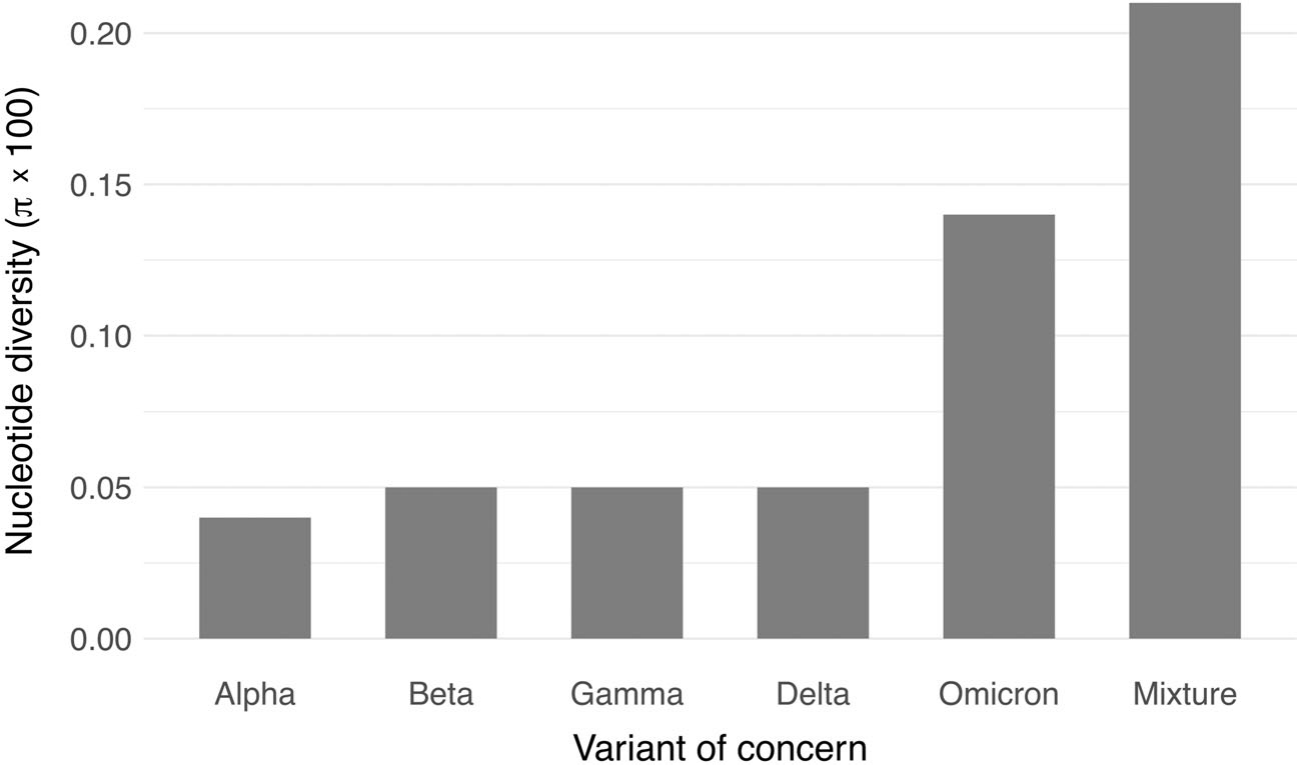
Nucleotide diversity in genome sequences from each variant of concern and combination of variants of concern. Nucleotide diversity (π) was calculated for the genome sequences of each variant of concern, as well as for a combination of variants of concern (labeled as Mixture).

**Figure 2:**
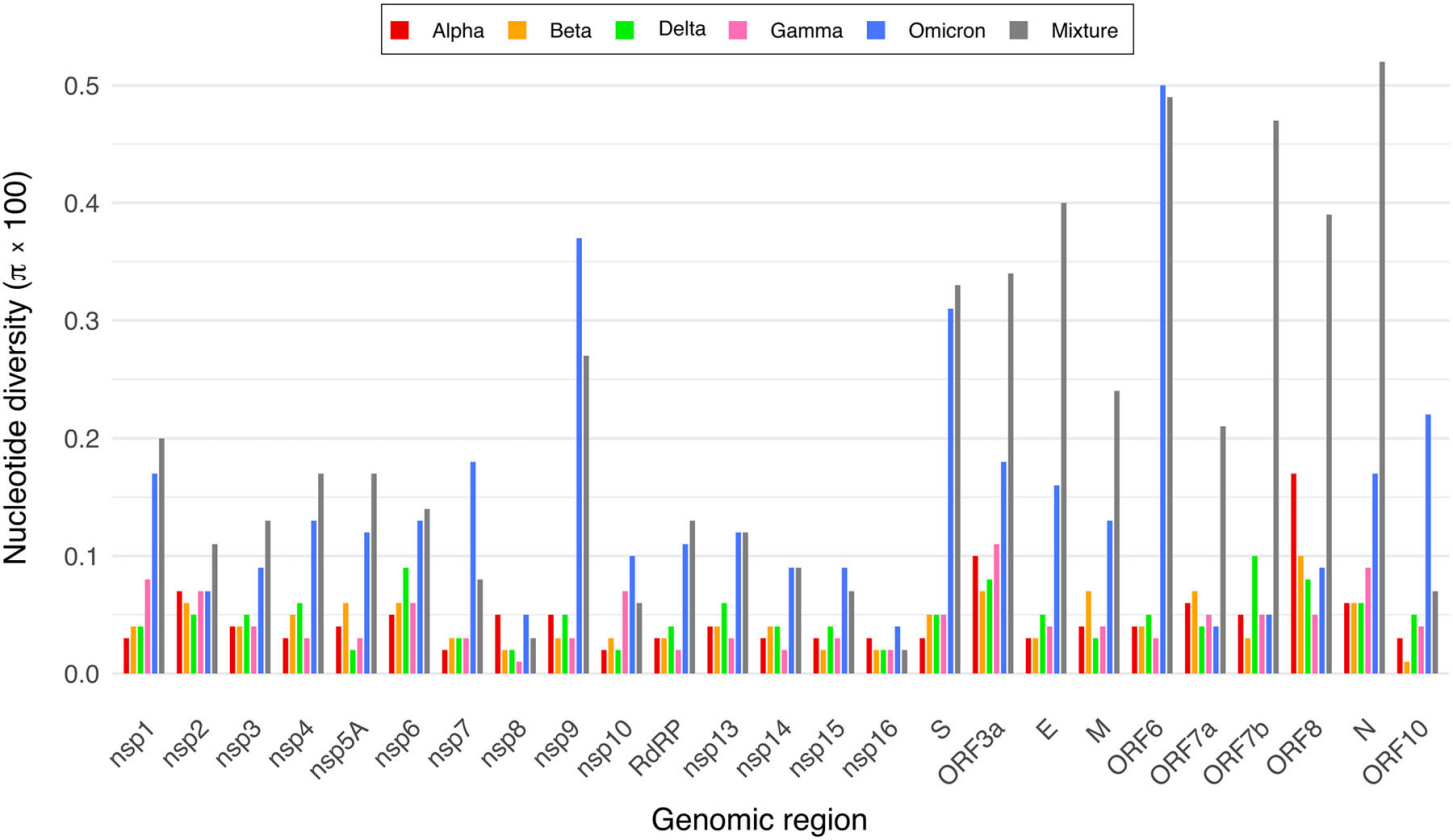
Nucleotide diversity for each coding region in each variant of concern and combination of variants of concern. Nucleotide diversity (π) was calculated for the 25 coding regions in the genome of each variant of concern, as well as for a combination of variants of concern (labeled as Mixture).

In general, the nucleotide diversity (π) was low and varied across the study data. Across the genome, we observed nucleotide diversity values ranging from approximately 0.04% to 0.21%, depending on the variant and region analyzed. Among the VOCs, at the genome‐wide level, the Alpha variant exhibited the indicated lowest nucleotide diversity, and the Omicron variant showed the highest nucleotide diversity, nearly three times higher than that of the other variants (Figure 1). The Beta, Gamma, and Delta variants exhibited low and similar nucleotide diversity, approximately 0.05%. When considering all the VOCs together, the observed nucleotide diversity (approximately 0.21%) was even higher than that of the Omicron variant (Figure 1). At the genomic region level, nucleotide diversity generally showed broad variations among different genomic regions (Figure 2), with values ranging from over 0.01% in conserved regions to over 0.5% in more variable genomic coding regions. For instance, the coding regions for nsp8 and nsp16 showed low levels of genetic diversity, even in the Omicron variant. In contrast, other regions (i.e., ORF7a, ORF7b, and ORF8) did not exhibit increased nucleotide diversity in the Omicron variant compared to the other VOCs, while other regions (i.e., nsp9, S, and ORF6) displayed high levels of diversity, especially in the Omicron variant (Figure 2).

When exploring the nucleotide diversity of all existing variants, not just the VOCs, we found some additional particularities at each time period. Specifically, we identified overall expansions in diversity during the early years of the pandemic (above 0.12%) and during the emergence of the Omicron variant (above 0.2%), followed by contractions in diversity (below 0.07%) (Supporting Information S1: Figure S2). Interestingly, these fluctuations also varied among coding regions (Supporting Information S1: Figures S3 and S4). Certain coding regions, particularly genes such as S, N and ORF6, exhibited higher diversity (above 0.15%) compared to other regions, especially nonstructural protein‐coding regions like nsp15 and nsp16 (Supporting Information S1: Figure S3). Most coding regions (i.e., nsp2, nsp3, nsp6, nsp7, nsp14, among others) followed the previously indicated pattern of temporal expansions and contractions of nucleotide diversity (Supporting Information S1: Figure S4). However, some coding regions (i.e., nsp5A and nsp15) displayed distinct patterns, such as expansions of diversity during time periods associated with the emergence of specific VOCs, such as Delta (Supporting Information S1: Figure S4).

### 3.2 General Lack of a Strict Molecular Clock, With Variations of the Rate of Evolution Both Over Time and Among Different Coding Regions

**Figure 3:**
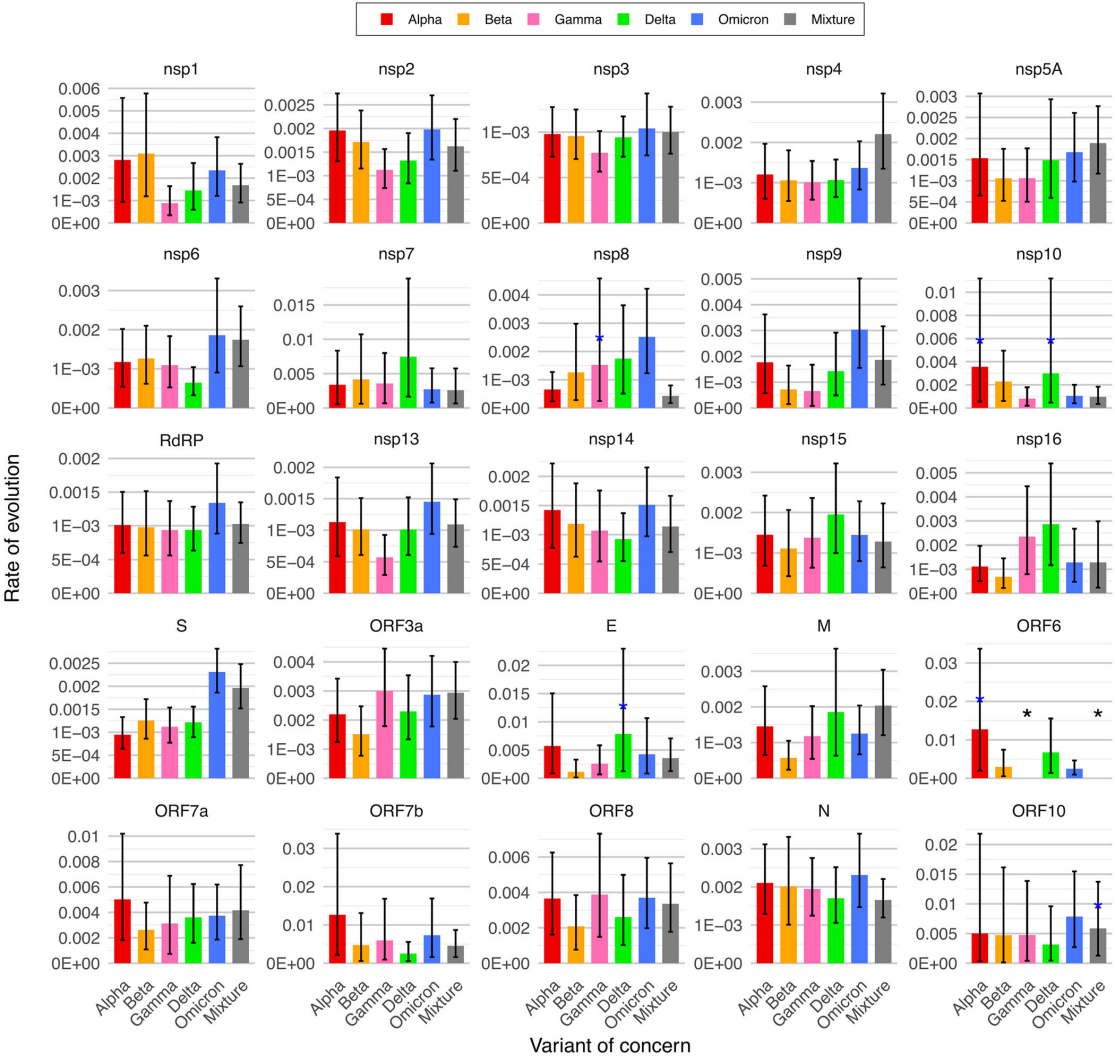
Rate of molecular evolution for each coding region in each variant of concern and combination of variants of concern. The rate of molecular evolution was estimated for each of the 25 coding regions in the genome of each variant of concern, as well as for a combination of variants of concern (labeled as Mixture). Error bars indicate the 95% highest posterior density interval (HPDI). Overall, the data converged with an effective sample size (ESS) above 200, but some estimates had an ESS below 200 (blue asterisk) or did not converge on the HPDI (black asterisk).

We found that the hypothesis of SARS‐CoV‐2 molecular evolution following the strict molecular clock was rejected (*p*‐value < 0.05) for all VOC data, including the mixed VOC data set. This rejection also applied to nearly all coding regions, such as nsp1, nsp2, nsp3, nsp4, nsp5A, nsp6, RdRP, nsp13, nsp14, nsp15, as well as the genes S, M, N, ORF3a, ORF6, and ORF7a. These findings suggested the use of relaxed molecular clock models to study the rate of evolution. We estimated an overall rate of evolution of 10^−3^ substitutions per site per year. When comparing the estimated rates of molecular evolution for each VOC data set separately and for the mixed VOC data set, we did not find significant differences (Supporting Information S1: Figure S5). Similar findings were found when considering all SARS‐CoV‐2 variants, not just the VOCs, sampled at different time periods (Supporting Information S1: Figure S6). However, we found variations in the estimated rate of molecular evolution among some coding regions (Supporting Information S1: Figure S7), with more pronounced differences in the VOCs data (Figure 3), and also noted in the temporal periods data (Supporting Information S1: Figure S8). For instance, we observed a significant increase in the rate of molecular evolution in the S gene of the Omicron variant compared to previous VOCs (Figure 3), a pattern not found in other genes, such as most of the nsp coding regions (Figure 3). We encountered difficulties in estimating the rate of evolution for certain coding regions of the early variants due to their low genetic diversity, as convergence of MCMC chains did not occur even after more than 125 M iterations. This aligns with findings in previous SARS‐CoV‐2 phylogenetic studies dealing with early data [37, 38].

### 3.3 Heterogeneous Molecular Adaptation Among SARS‐CoV‐2 Coding Regions and Over Time

**Figure 4:**
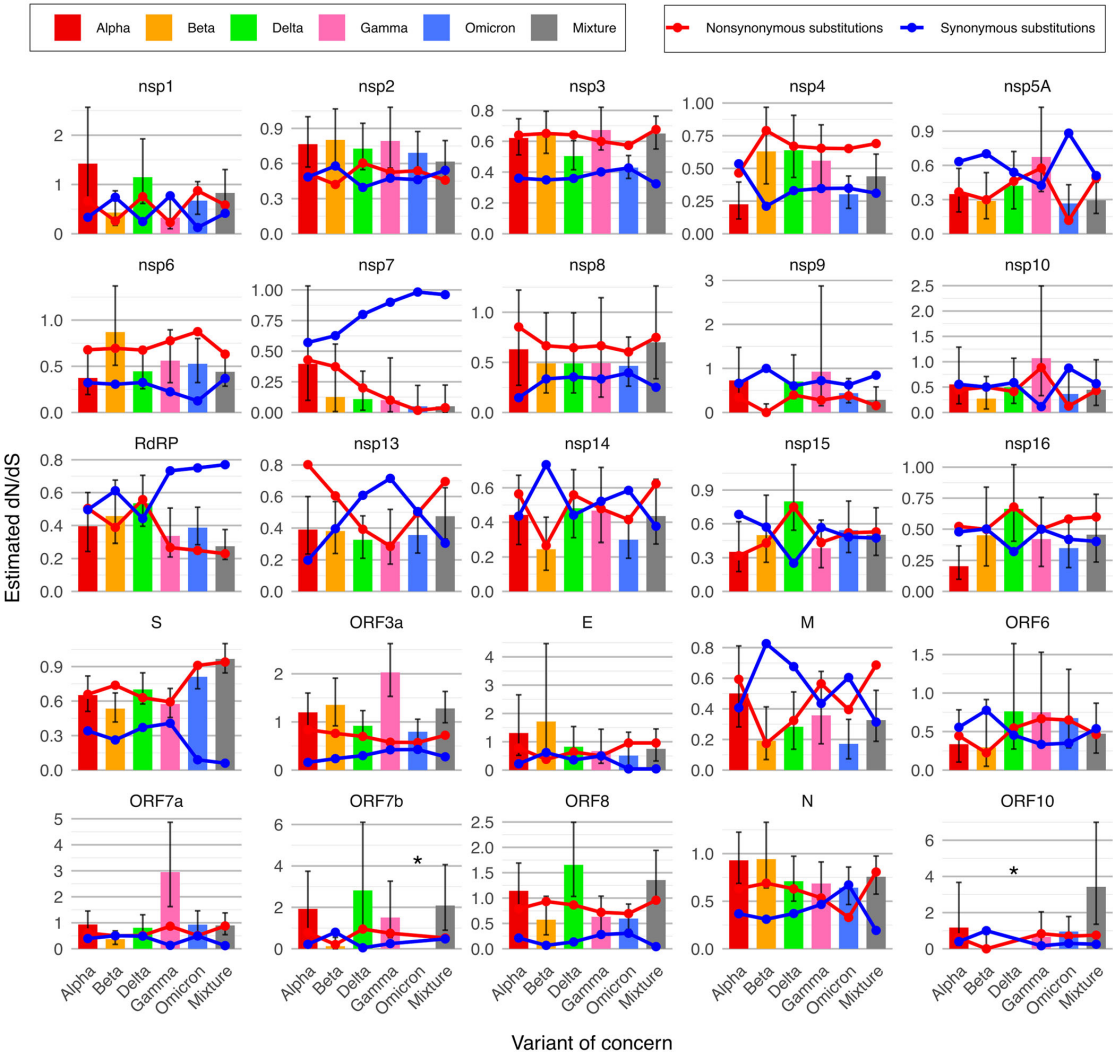
Selective pressure detected in each coding region of each variant of concern and combination of variants of concern. For each coding region, the bars indicate the estimated nonsynonymous synonymous substitution rate ratio (*dN/dS*) from each variant of concern and for the combination of variants of concern (labeled as Mixture). The error bars indicate the 95% confidence interval provided by the estimation method. Additionally, each plot includes the observed proportion of synonymous (blue line) and nonsynonymous (red line) codon changes. Estimates that were not available (i.e., due to insufficient observed codon changes) are indicated with asterisks.

We found that the global molecular adaptation (estimated from all coding regions) for each VOC showed evidence of purifying selection, with no significant differences among the VOCs (Supporting Information S1: Figure S9). However, we observed notable variations of molecular adaptation among coding regions and over time (Figure 4). For instance, we identified positive selection in the ORF3a and ORF7a genes of the Gamma variant and in the ORF8 gene of the Delta variant (Figure 4). Interestingly, we did not observe a general increase in diversifying adaptation in the coding regions of the Omicron variant, compared to earlier VOCs, except for the S gene. To further explore these findings, we investigated the number and type of substitutions observed in each coding region and across different time periods. Firstly, we found that the number of nonsynonymous codon changes exceeded the number of synonymous codon changes, considering all the SARS‐CoV‐2 coding regions for each VOC (Supporting Information S1: Figure S10). Importantly, we also found that the Omicron variant accumulated a higher number of both nonsynonymous and synonymous codon changes compared to the earlier VOCs. In general, these findings were also observed in the separate analysis of each gene (Supporting Information S1: Figures S11 and S12), although in a few genes (i.e., M), the number of synonymous codon changes was higher than the number of nonsynonymous codon changes in some VOCs. Regarding the S gene, we found a large number of nonsynonymous codon changes in the Omicron variant compared to the corresponding synonymous codon changes (Supporting Information S1: Figure S11).

Considering data from all SARS‐CoV‐2 variants, not just the VOCs, we observed general fluctuations in molecular adaptation over time across all the studied coding regions (Supporting Information S1: Figure S13), with a slight increase during part of 2021, followed by a decrease in 2022 and a subsequent increase in 2023. This finding is associated with the corresponding number of synonymous and nonsynonymous codon changes observed during those periods of time (Supporting Information S1: Figure S14). At the coding region level, most coding regions exhibited overall purifying selection (Supporting Information S1: Figure S15). However, their selection pressures varied over time and across different coding regions (Supporting Information S1: Figure S16). Some regions, such as the *S* gene, showed an increase in diversifying selection over time, while others, such as nsp2, nsp3, nsp13, and N, exhibited increased purifying selection over time. Additionally, coding regions such as nsp14 and ORF8 displayed fluctuating selection pressures over time. Again, this finding is associated with the corresponding number of synonymous and nonsynonymous codon changes observed during those time periods (Supporting Information S1: Figures S17 and S18). For instance, we found a large number of nonsynonymous substitutions, compared to the respective synonymous substitutions, observed in the S gene (Supporting Information S1: Figures S17 and S18). We also examined selection within each coding region and found several significant PSSs (Supporting Information S1: Table S1) and a large number of significant NSSs (Supporting Information S3: Table S3) across all coding regions. Thus, although most genes and codon sites in SARS‐CoV‐2 evolved under purifying selection, some functionally relevant regions showed recurrent signatures of diversifying selection across data sets. Notably, we found that both PSSs and NSSs tended to occur frequently across data sets of different temporal periods and VOCs within the S1 subunit of the spike protein (Supporting Information S1: Figure S19). In this regard, in the S gene, the PSSs detected across different VOCs were located in the N‐terminal domain (NTD) and the receptor binding domain (RBD) (Supporting Information S1: Figure S19). Indeed, analyzing each VOC separately could reduce the number of detected PSSs compared to considering all coexisting variants together. In fact, the selection analysis of the S gene using data from all coexisting variants revealed PSSs in 15 residues of the NTD, 9 residues of the RDB, and 2 residues of the heptad repeat 1. In this data, we found PSSs in residues that do not define VOCs (i.e., residues 16, 23, 51, 52, 68, 14, 248, 402, 413, 444, 450, 462, 481, 482, 936, and 937), in residues used to define Omicron sublineages (i.e., residues 24, 27, 143, 211, 375), and in residues shared by multiple VOCs (i.e., residue 26, shared by Gamma and Omicron subvariants; residues 69, 70, and 144 shared by Alpha and Omicron subvariants; residue 484, shared by Beta, Gamma, and Omicron subvariants).

## 4 Discussion

Despite a general perception that SARS‐CoV‐2 is an issue of the past, this virus continues to circulate, affecting public health systems worldwide with ongoing infections and deaths, particularly among vulnerable and elderly people. Indeed, uncertainty remains regarding the potential emergence of new SARS‐CoV‐2 variants that may evade current therapies. Actually, current vaccines have shown reduced efficacy against circulating Omicron variants [39], indicating the need for vaccine updates that account for the molecular evolution of the virus. In this regard, understanding the molecular evolution of SARS‐CoV‐2 is crucial for predicting future variants. Previous studies exploring the molecular evolution of this virus have often relied on relatively small data, data collected from the same time period, or, more importantly in our view, have overlooked potential variations in evolutionary patterns across coding regions and over time. Thus, we conducted a comprehensive study examining molecular diversity, the rate of evolution, and molecular adaptation in the different SARS‐CoV‐2 coding regions (which are crucial for vaccine development) using a large data set of genome sequences of the diverse variants and sampled at different temporal periods. Instead of analyzing a single data set of current sequences and inferring their past evolution, we leveraged the extensive collection of genome sequences, sampled across different time periods and associated with various variants, to study molecular evolution across different VOCs and temporal periods. This approach reduces prediction errors by incorporating real data from each temporal period. Although our analyses were still constrained by data size due to computational limitations and the inherent simplifications of evolutionary models, we believe this study provides a robust approximation of the molecular evolution of the virus. We show that the evolutionary patterns of SARS‐CoV‐2 vary among coding regions and over time (as discussed further below), and these findings suggest that predicting future variants is more complex than expected.

In general, the genetic diversity in SARS‐CoV‐2 is low, although it has increased over time with the emergence and establishment of new variants. We observed fluctuations in the overall genetic diversity over time. In particular, low diversity was observed at the start of the pandemic, likely due to the recent introduction of the virus and insufficient time for the accumulation of mutations [40, 41]. This was followed by a gradual increase in diversity, related to the appearance and concurrent spread of various variants, not always originating from the same lineages [6], with a more significant rise during the emergence of the Omicron variant. Counterintuitively, we also observed decreases in genetic diversity after the establishment of certain variants, which could be explained by the replacement of previous variants as a single variant becomes predominant [42]. For example, since the end of 2021, various subvariants of Omicron have replaced one another over time [42], leading to fluctuations in overall genetic diversity. This pattern initially showed an increase in diversity (due to the confluence of multiple subvariants), followed by a decrease, such as the replacement of most of the subvariants by the JN.1 Omicron subvariant [42].

At the genomic region level, although most genomic regions showed a sharp increase in diversity in Omicron compared to earlier VOCs, some genomic regions maintained their diversity over time, including in Omicron. These findings indicate a spatial and temporal heterogeneity in the molecular diversity of SARS‐CoV‐2, which we interpret as a consequence of variable selective pressures among coding regions (i.e., genes related to virus transmission may be less conserved to improve their phenotypic efficiency) and over time (i.e., optimizing efficiency compared to earlier variants, with new SARS‐CoV‐2 variants rapidly replacing their predecessors). For example, we detected an increase in diversity through multiple nonsynonymous codon changes in the gene encoding the spike protein of the Omicron variant, which could facilitate immune evasion and rapid spread [43]. We also observed a significant increase in diversity in ORF6, a gene that inhibits the nuclear translocation of STAT1 and STAT2, exerting an anti‐IFN activity [44]. This increased variability could enhance its ability to inhibit IFN signaling and facilitate immune escape. For some coding regions (i.e., nsp9 [45]), we detected an increase in nucleotide diversity, mainly driven by synonymous codon changes, which would likely not affect the corresponding protein activity. In addition to selection pressures from immunity, we consider that other factors, such as public health measures including lockdowns, curfews, and movement restrictions, may have contributed to the observed fluctuations in viral genetic diversity [46, 47]. It is important to take into account that the genetic diversity observed at each genomic region, in light of its variability among genomic regions, can inform genomic surveillance studies (e.g., S gene, ORF6, ORF3a, and ORF7a) [48], as well as the development of pan‐variant diagnostics and antiviral therapies targeting more conserved regions (e.g., nsp8, nsp16) [49, 50, 51]. For instance, we found that the nsp5 region is well conserved, and therefore its encoded protein Mpro may be a potential target for antiretroviral drugs, as previously proposed [52, 53]. The N protein may also be considered a potential target, given its observed genetic conservation, as also previously suggested [54, 55].

Regarding the rate of molecular evolution, the strict molecular clock hypothesis was rejected for all VOC data sets. Several factors could explain this heterogeneity, such as varying immune pressures among VOCs and population bottlenecks during periods of dominance of specific variants [56]. Indeed, other studies also found deviations from the strict molecular clock [29, 57, 58], suggesting evolutionary accelerations associated with the emergence of variants with higher transmissibility and immune evasion, which aligns with the violation of the molecular clock observed in our mixed VOC data. Altogether, the disruption of the strict molecular clock is driven not only by the emergence of new variants and lineages but also by subsequent factors, such as changes in population size. However, further work is needed to test these potential causes formally. At the coding region level, which, to our knowledge, has not been previously investigated, we also observed heterogeneity. For instance, the structural proteins S, M, and N exhibited notable changes over time in the rate of evolution that did not follow the strict molecular clock, likely due to selective pressures from the host environment. The disruption of the molecular clock was also observed in most of the nonstructural protein‐coding regions of the ORF1ab gene, which are primarily responsible for the viral replication [59], also likely due to adaptation to new host environments. In the genes ORF3a, ORF6, and ORF7a, the variation in the rate of evolution over time could also be attributed to adaptations to the host immune system, as well as improved viral replication and transmission, among other factors [44, 60, 61]. For some coding regions with smaller sizes, such as the one encoding the E protein, we encountered difficulties in estimation due to low genetic diversity. Given our findings on the widespread poor fit of the strict molecular clock with SARS‐CoV‐2 genetic data, we recommend that future evolutionary analyses of SARS‐CoV‐2 consider using relaxed clock models better to capture rate variation across lineages and genomic regions.

We estimated an overall rate of evolution on the order of 10^−3^ substitutions per site per year, which is consistent with previous estimates for SARS‐CoV‐2 [12] and other coronaviruses [6]. The lack of significant differences in the mean rate of gene evolution among VOCs suggests that, while the rate of evolution varied among certain genomic regions, the overall average remained relatively constant. Interestingly, the rate of evolution in the *S* gene increased in the Omicron variant compared to earlier variants, which may reflect adaptive shifts with potential phenotypic consequences, such as increased transmissibility or immune escape. This highlights the need for continued monitoring of this genomic region, particularly in emerging sublineages. Also, several coding regions (including the structural genes S, M, N, E, the ORF3a and ORF7a genes, and the regions encoding nsp3, nsp5A, nsp6, nsp10, and RdRP) exhibited a progressive increase in the rate of evolution over time, culminating in the Omicron variant, likely driven by increased fitness for adaptation.

As previously shown, the observed genetic diversity and rate of evolution can be related to molecular adaptation. In this context, given that genetic diversity and rate of evolution varied among coding regions and over time, we investigated selection in the variety of SARS‐CoV‐2 coding regions. As expected, purifying selection was detected in all the coding regions, consistent with previous studies [16, 62, 63]. This can be explained by the fact that, since the start of the pandemic, the virus has shown a high level of adaptation to humans [64], likely facilitated by possible previous interactions [65]. Notably, estimating the temporal dynamics of selection enables understanding of whether specific selective pressures are changing or being maintained over time, providing insights that can inform variant surveillance and the design of updated therapies [66, 67]. Interestingly, as found with the previously discussed evolutionary patterns, the detected selection pressures varied among coding regions and over time. We detected a trend toward an increasing *dN/dS* ratio in the Omicron variants of the S gene compared to earlier variants, which is expected given its role in the immune response and its use as a target for vaccines and antibody therapies [43]. In other coding regions, the estimated *dN/dS* remained constant or showed slight fluctuations over time. This was unexpected given the higher genetic diversity generally observed in Omicron variants. To understand this lack of correlation, we thoroughly investigated the number of synonymous and nonsynonymous codon changes present in each data set. We found that, in many coding regions, genetic diversity increased due to a relatively high proportion of synonymous changes compared to nonsynonymous changes (notice that, by chance, nonsynonymous codon changes are more than twice as likely to occur as synonymous ones). Therefore, genetic diversity mainly increased at the nucleotide level rather than at the amino acid level. Next, we detected an increase in purifying selection over time in the N gene and some ORF1ab coding regions, which may suggest the maintenance of certain functional properties, such as immune escape and optimized processes of viral replication and assembly [68]. Similarly, the overall maintenance of selection in the region encoding the M protein could be related to its essential role in virion assembly [69, 70]. We also observed fluctuations in selection over time in ORF3a, ORF7a, and ORF8, which may reflect episodes of fitness maintenance and improvement through interactions with immune hosts. In particular, these three proteins are crucial for immune evasion, inflammation induction, and the alteration of essential cellular processes [71, 72, 73], making their selected variants potentially advantageous for viral replication and spread. Next, as expected from the overall purifying selection, we identified a large number of NSSs, likely associated with maintenance of protein stability and activity [74, 75]. However, we also detected several PSSs within coding regions, which could result from molecular adaptation at specific protein sites, likely aimed at increasing fitness. In the S gene, the PSSs detected across different VOCs were mainly located in the NTD and the RBD (Supporting Information S1: Figure S20), probably due to increased viral fitness considering the major functional importance of these protein regions. In particular, the RBD is crucial for binding to the human angiotensin‐converting enzyme 2 (ACE2), and the affinity between both protein regions determines viral infectivity and transmissibility. Moreover, the RBD is the main target of vaccines and antibody therapies [76]. The NTD, on the other hand, binds to molecular components on the surface of the host cell, facilitating viral attachment to the cell and complementing the function of the RBD [77]. Some studies suggested that the NTD can be recognized by specific monoclonal antibodies, and mutations in this region can facilitate immune escape [78]. Interestingly, some of the detected PSSs defined the VOCs in which they were found. Specifically, in the S gene, amino acid changes at residues 24 and 27 characterize the Omicron BA.2 subvariant, changes at residues 70 and 243 define the Beta variant, changes at residues 142 and 143 are associated with the Omicron BA.1 subvariant, and changes at residue 142 also define the BA.4 and BA.5 subvariants [79]. Notably, we detected a PSS at residue 143, involving an amino acid change used to define Omicron subvariants, already during the period of Alpha variant prevalence. The early appearance of this site under positive selection indicates the importance of temporal selection analyses to anticipate evolutionary pathways before they become epidemiologically dominant. Such early molecular signatures could also contribute to improving variant monitoring and forecasting, as well as inform timely updates of therapeutic strategies such as vaccines. We expected to find additional PSSs, especially in the Omicron data and in the data combining VOCs, but many potential PSSs were not statistically significant. Next, we found that the number of PSSs was lower when analyzing each VOC separately than when considering all coexisting variants together, which can be expected since this data considers a larger evolutionary time. Multiple detected PSSs can be related to functional molecular adaptations. For example, at the S gene, multiple PSSs that appeared repeatedly across temporal periods and VOCs are located within fundamental regions involved in the fusion of the viral membrane with the host cell, such as the S1 subunit [80]. This region is exposed on the viral surface and is directly involved in host receptor recognition, making it more susceptible to immune pressure and functional adaptation. These findings are also consistent with previous observations where surface‐exposed and immunologically relevant regions tend to evolve under a combination of diversifying and purifying constraints [47]. Temporal shifts in selection in these regions may also reflect lineage‐specific adaptations or immune‐driven diversification. Altogether, we detected heterogeneity among coding regions and over time in terms of local selection, with some coding regions showing no PSS and others displaying a variety of PSSs, which varied over time. In general, the results indicated a general evolutionary process driven by purifying selection, with certain local changes contributing to improved molecular fitness.

The interplay between different evolutionary mechanisms, including the generation of new molecular variants subject to selection, that produces the observed molecular diversity can provide valuable insights for monitoring and genomic surveillance strategies, as well as for the design and updating of therapies [49, 81]. Relationships between observed nucleotide diversity and molecular adaptation are not straightforward, as various types of selection can occur across different levels of nucleotide diversity. For instance, increasing nucleotide diversity should not be interpreted as diversifying selection, as most nucleotide changes could be silent (synonymous) for the protein [34]. However, monitoring proteins with high variability (e.g., S, ORF6, ORF3a, and ORF7a) can be important to detect emerging mutations of concern, and identifying conserved proteins is important for designing diagnostic and therapeutic strategies since they are less prone to incorporating escape mutations and thus offer more reliable targets for pan‐variant interventions (e.g., nsp8, nsp16). In this regard, the *S* gene in the Omicron variant exhibited high genetic diversity and an increased rate of evolution, alongside strong signatures of diversifying selection and a predominance of nonsynonymous changes. These findings are consistent with the functional relevance of the spike protein in receptor binding, immune escape, and transmissibility [82]. The elevated rate of evolution and diversity in this region likely contributed to the rapid adaptive success of Omicron, allowing it to evade pre‐existing immunity and spread efficiently across diverse host populations. In contrast, ORF6 exhibited high levels of genetic diversity over time, alongside an overall neutral evolution pattern and a comparable number of synonymous and nonsynonymous substitutions. These findings suggest that mutations may accumulate in this protein without substantially affecting its function, which includes antagonizing type I interferon signaling and contributing to immune evasion [83]. Other possible mechanisms underlying this pattern can be compensatory evolution and constraints imposed by RNA secondary structures. Incorporating region‐specific evolutionary patterns into future studies would help refine early detection of relevant fixed mutations and support the design of robust diagnostic tools and therapies [12, 40].

Altogether, we found that SARS‐CoV‐2 evolves with high heterogeneity among its various coding regions and over time. In general, it did not follow the strict molecular clock, and genetic diversity fluctuated both among regions and over time. The recent Omicron subvariants showed an increase in the rate of evolution and genetic diversity, but this was observed only in the coding regions directly involved in virus transmission and replication. Heterogeneity was also observed in terms of selection, where, despite an overall pressure of purifying selection, local adaptations were established and replaced over time in certain genes, also directly related to transmission and replication in changing hosts. We emphasize that, to predict the future molecular evolutionary trajectories of this virus, as some studies investigate [84], the spatial and temporal heterogeneity of evolutionary processes observed in SARS‐CoV‐2 must be considered. In general, we recommend continuing to monitor and understand SARS‐CoV‐2 molecular evolution to detect new variants that could be significant in terms of disease severity or vaccine escape, as well as to anticipate the future outcome of this virus.

## Author Contributions

M.A. and L.D.G.‐V. conceived and designed the study. L.D.G.‐V., M.A., and D.F. developed analytical methods. L.D.G.‐V., P.I.‐R., and D.F. performed the analyses. All the authors wrote the manuscript and approved the final version of the manuscript.

## Conflicts of Interest

The authors declare no conflicts of interest.

## Supporting information


**Figure S1:** Phylogenetic tree derived from the dataset with genome sequences from different VOCs. **Figure S2:** Nucleotide diversity at each studied temporal period for the SARS‐CoV‐2 coding regions (average). **Figure S3:** Nucleotide diversity for each SARS‐CoV‐2 coding region across all studied temporal periods (average). **Figure S4:** Nucleotide diversity for each SARS‐CoV‐2 coding region at each studied temporal period. **Figure S5:** Average rate of molecular evolution for coding regions in each variant of concern and combination of variants of concern. **Figure S6:** Average rate of molecular evolution for coding regions across each studied temporal period. **Figure S7:** Rate of molecular evolution in each coding region considering all studied temporal periods. **Figure S8:** Rate of molecular evolution of each coding region at each studied temporal period. **Figure S9:** Selective pressure detected in average from the coding regions in each variant of concern and combination of variants of concern. **Figure S10:** Number of synonymous and nonsynonymous codon changes observed in the coding regions at each variant of concern and combination of variants of concern. **Figure S11:** Number of synonymous and nonsynonymous codon changes observed in each coding region of each variant of concern and combination of variants of concern. **Figure S12:** Number of synonymous and nonsynonymous codon changes observed in each non‐structural protein coding region of ORF1ab at each variant of concern and combination of variants of concern. **Figure S13:** Selective pressure detected on average from the coding regions in each studied temporal period. **Figure S14:** Number of synonymous and nonsynonymous codon changes observed in the coding regions at each studied temporal period. **Figure S15:** Selective pressure detected in each coding region considering all the studied temporal periods. **Figure S16:** Selective pressure detected in each coding region at each studied temporal period. **Figure S17:** Number of synonymous and nonsynonymous codon changes observed in each coding region considering all the studied temporal periods. **Figure S18:** Number of synonymous and nonsynonymous codon changes observed in each studied temporal period. **Figure S19:** Distribution of codon site‐specific selective pressure along the SARS‐CoV‐2 spike gene. **Figure S20:** Significant positively selected sites identified in the SARS‐CoV‐2 Spike protein across different variants of concern.

**Table S1:** Coding regions with PSSs in a variant of concern or during a specific temporal period. **Table S2:** Metadata for all SARS‐CoV‐2 sequences analysed in the temporal datasets.

**Table S3:** Coding regions with negatively selected sites (NSSs) in all the study data.

## Data Availability

The data that support the findings of this study are openly available in Zenodo at https://doi.org/10.5281/zenodo.14981416.
