# Supplementary material for: Heterogeneous Evolution Among SARS‐CoV‐2 Genes and Variants of Concern
Source: J Med Virol. 2025 Sep 12;97(9):e70604. doi: 10.1002/jmv.70604 (PMC12431720; doi:10.1002/jmv.70604)

Supplementary Material

Heterogeneous evolution among SARS-CoV-2 genes and variants of concern

The supplementary material includes the Table S1 and Figures S1-S20.

Table S1. Coding regions with PSSs in a variant of concern or during a specific temporal period. Significant PSSs were identified with a *p*-value < 0.05.

VOC or temporal period	Coding region (length in codons)	Positively selected codon site
Alpha	S (1280)	143, 70, 243
	ORF7a (123)	4
Beta	S (1274)	243
Gamma	ORF6 (74)	58
Delta	S (1290)	247, 52, 141, 142
	ORF7a (198)	39
Omicron	S (1369)	485, 454, 143, 24, 27, 124
	ORF8 (128)	92
	N (420)	33
VOCs mixture	S (2110)	142, 144, 211
	ORF6 (75)	58
	ORF8 (128)	92, 119
09/2020 – 12/2020	S (1283)	51
01/2021 – 04/2021	S (1280)	70, 144
	ORF6 (82)	33
05/2021 – 08/2021	S (1283)	52, 141, 143
	nsp15 (348)	31
09/2021-12/2021	S (1295)	52, 69, 141, 144
	ORF3a (279)	103
	nsp6 (290)	107
01/2022 – 04/2022	nsp6 (291)	37, 106
	nsp3 (1945)	1266
	N (420)	33
05/2022 – 08/2022	S (1293)	24, 27, 68, 70, 248, 375, 450
	N (429)	33
09/2022 – 12/2022	S (1274)	27, 70
	N (429)	33
01/2023 – 04/2023	S (1288)	24, 27, 70, 144, 248, 444, 462, 484
	nsp4 (501)	64
	nsp2 (639)	97
	nsp14 (528)	430
	N (420)	33
05/2023 – 08/2023	S (1277)	27, 144
	ORF8 (126)	92
	ORF10 (53)	3
	nsp3 (1945)	1001
	N (420)	33
09/2023 – 12/2023	S (1329)	16, 26, 69, 143, 402, 413, 481, 936
	ORF3a (279)	103
	N (420)	33
	M (223)	30
01/2024 – 04/2024	S (1287)	16, 23, 26, 69, 143, 211, 402, 482, 937
	ORF8 (126)	92
	N (420)	33

Figure S1. Phylogenetic tree derived from the dataset with genome sequences from different VOCs. Maximum likelihood phylogenetic tree based on the corresponding best-fitting substitution model of DNA evolution inferred from the dataset (labeled as "Mixture") with genome sequences of different VOCs. The tree was rooted according to the known the temporal distribution of VOCs. Genome sequences were properly clustered according to their respective VOCs.

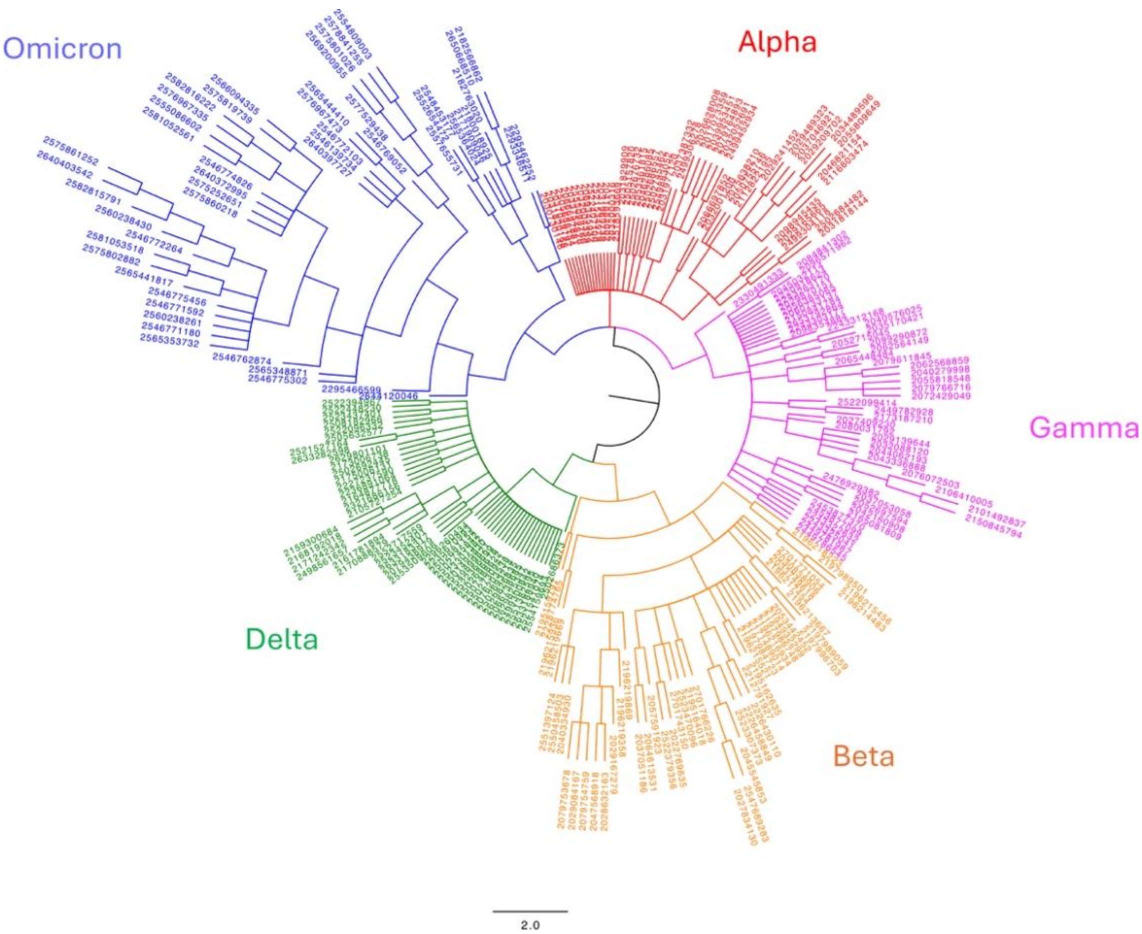

Figure S2. Nucleotide diversity at each studied temporal period for the SARS-CoV-2 coding regions (average). Average nucleotide diversity (π) of the 25 studied SARS-CoV-2 coding regions, weighted according to the length of the corresponding coding region, over time.

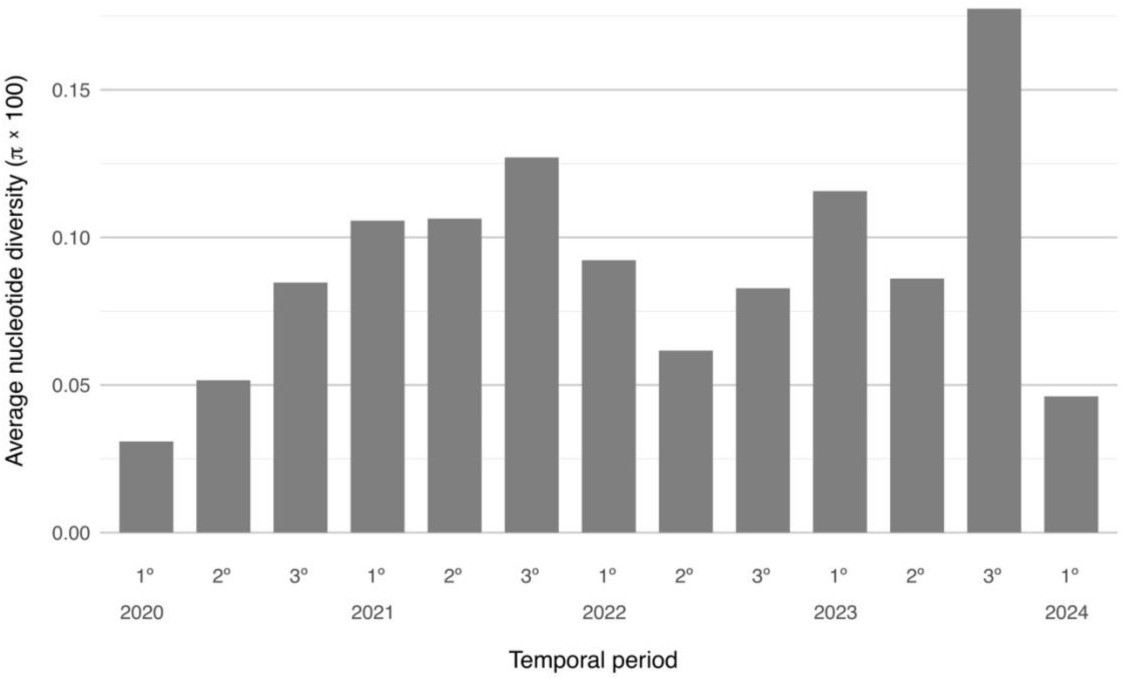

Figure S3. Nucleotide diversity for each SARS-CoV-2 coding region across all studied temporal periods (average). Average nucleotide diversity (π) for each SARS-CoV-2 coding region considering data from all the studied temporal periods.

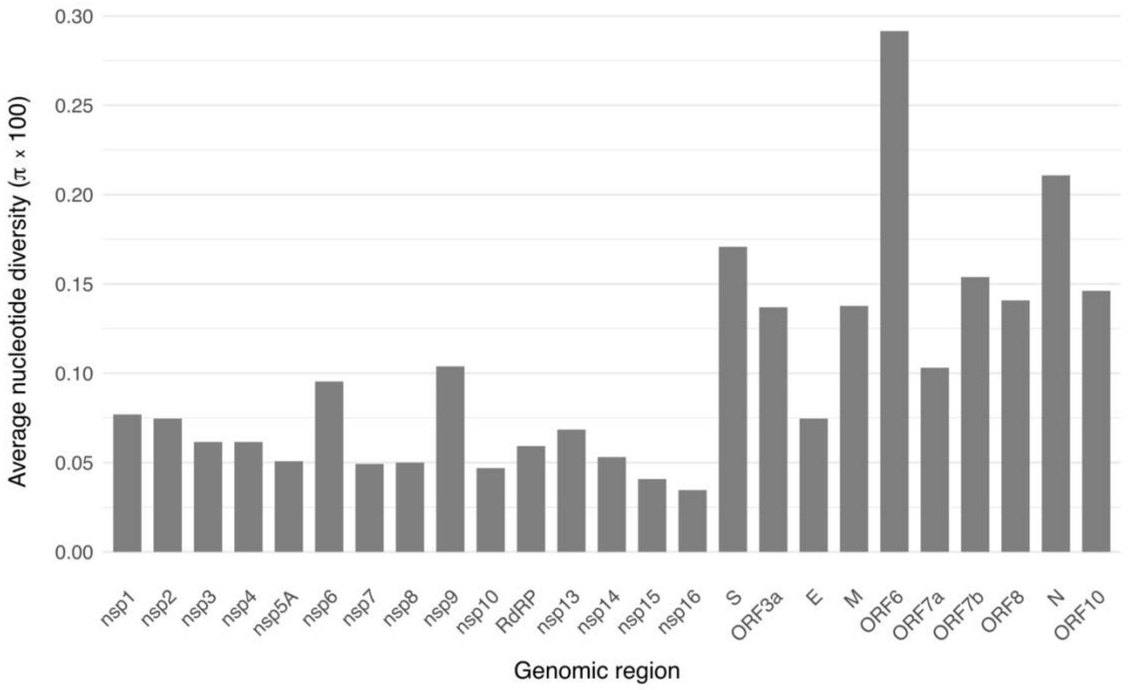

Figure S4. Nucleotide diversity for each SARS-CoV-2 coding region at each studied temporal period. Nucleotide diversity (π) for each SARS-CoV-2 coding region at each studied temporal period.

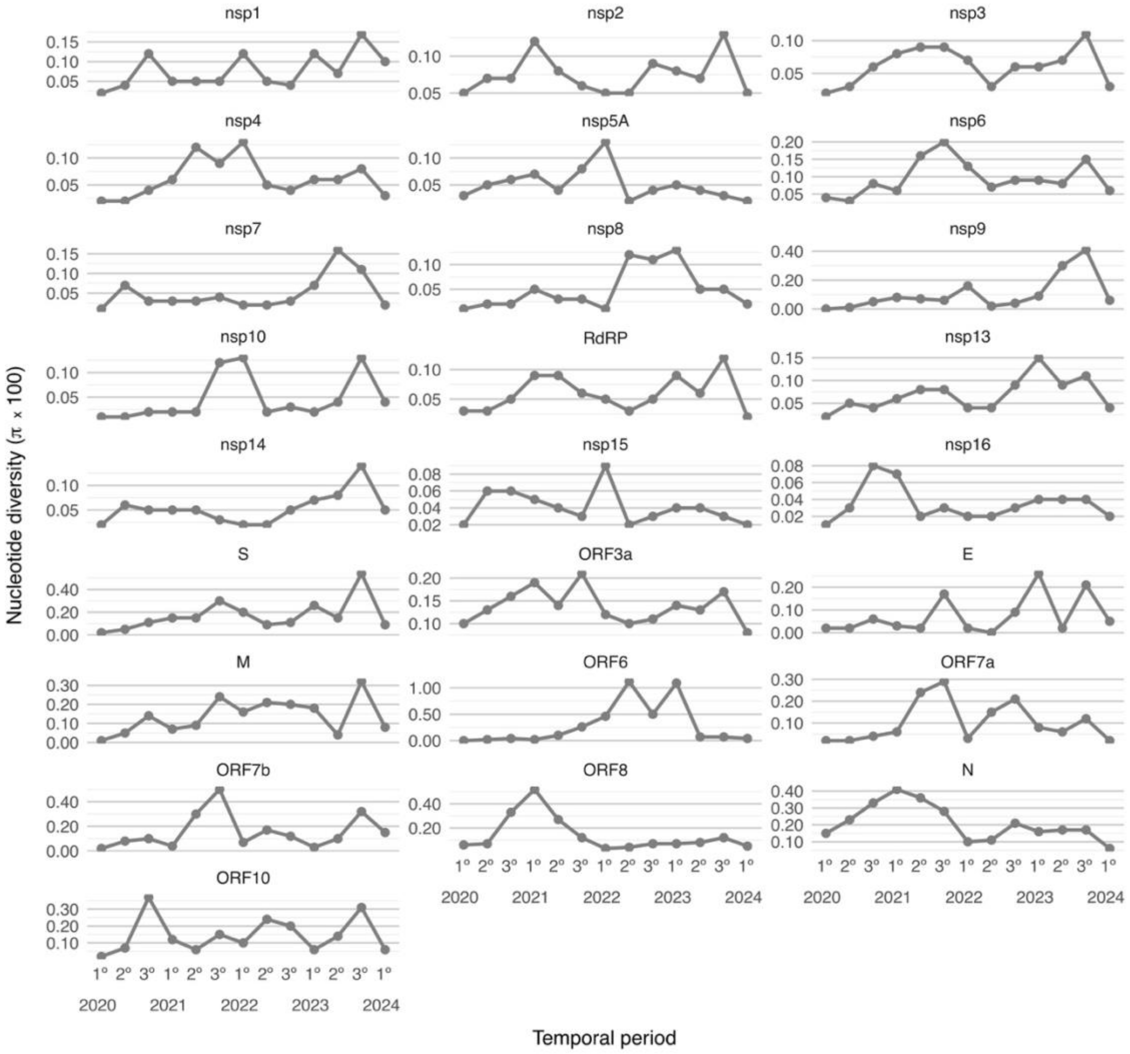

Figure S5. Average rate of molecular evolution for coding regions in each variant of concern and combination of variants of concern. For each variant of concern and for a combination of variants of concern (labeled as "Mixture"), the rate of evolution for each of the 25 coding regions is presented in average. Error bars indicate the 95% highest posterior density interval (HPDI), calculated as the mean of the HPDIs for all coding regions, weighted by the length of each coding region. Unconverged estimates were excluded (Figure 3). Detailed estimates for each coding region within each VOC are shown in Figure 3.

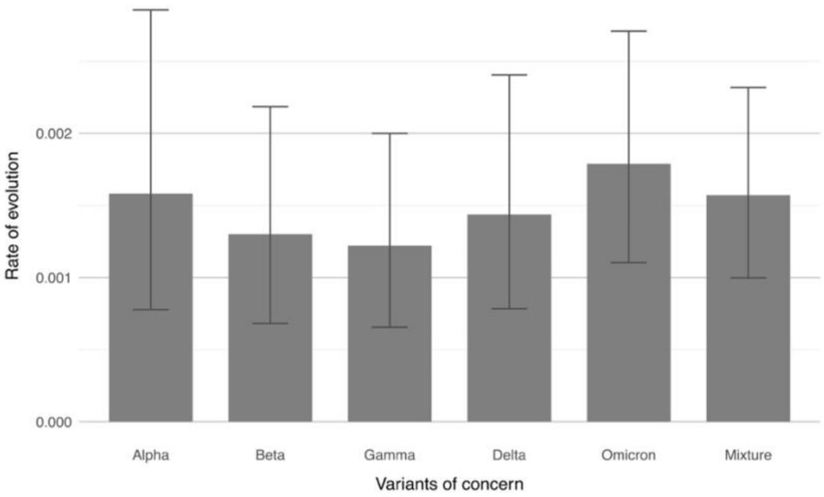

Figure S6. Average rate of molecular evolution for coding regions across each studied temporal period. The average rate of evolution for each of the 25 coding regions is presented for each studied temporal period. Error bars indicate the 95% highest posterior density interval (HPDI), calculated as the mean of the HPDIs from all 25 coding regions, weighted by the length of the corresponding coding regions. Unconverged estimates were excluded (Figure S8). Detailed estimates for each coding region within each temporal period are shown in Figure S8.

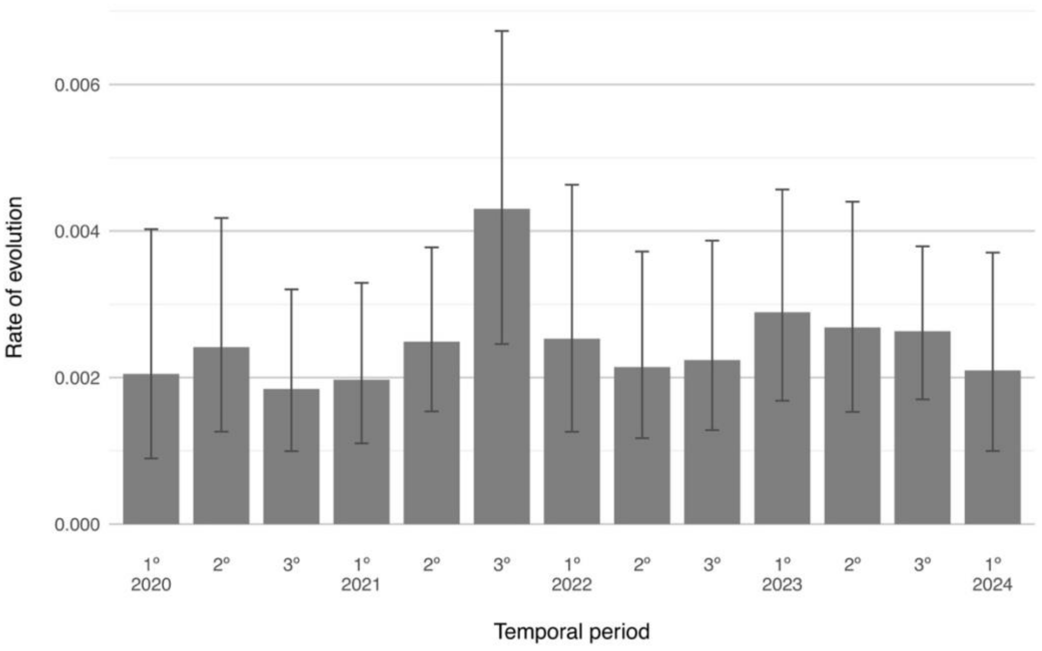

Figure S7. Rate of molecular evolution in each coding region considering all studied temporal periods. The rate of evolution for each of the 25 coding regions is presented as an average considering all the studied temporal periods. Error bars represent the 95% highest posterior density interval (HPDI), calculated as the mean of the HPDIs from the estimates at each temporal period. Unconverged estimates were excluded (Figure S8). Detailed estimates for each coding region at each studied temporal period are shown in Figure S8.

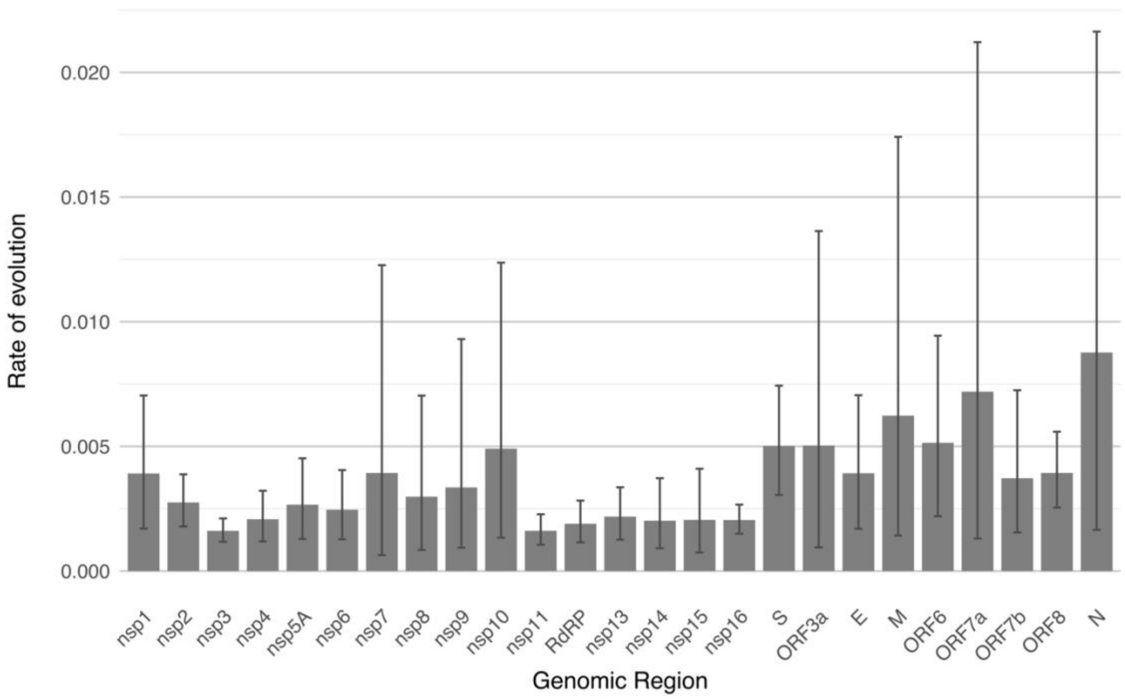

Figure S8. Rate of molecular evolution of each coding region at each studied temporal period. The rate of evolution for each of the 25 coding regions is presented for each studied temporal period. Error bars represent the 95% highest posterior density interval (HPDI). Overall, the data converged with an effective sample size (ESS) above 200, but some estimates did not reach an ESS of 200 (blue asterisk) or did not converge the HPDI (black asterisk).

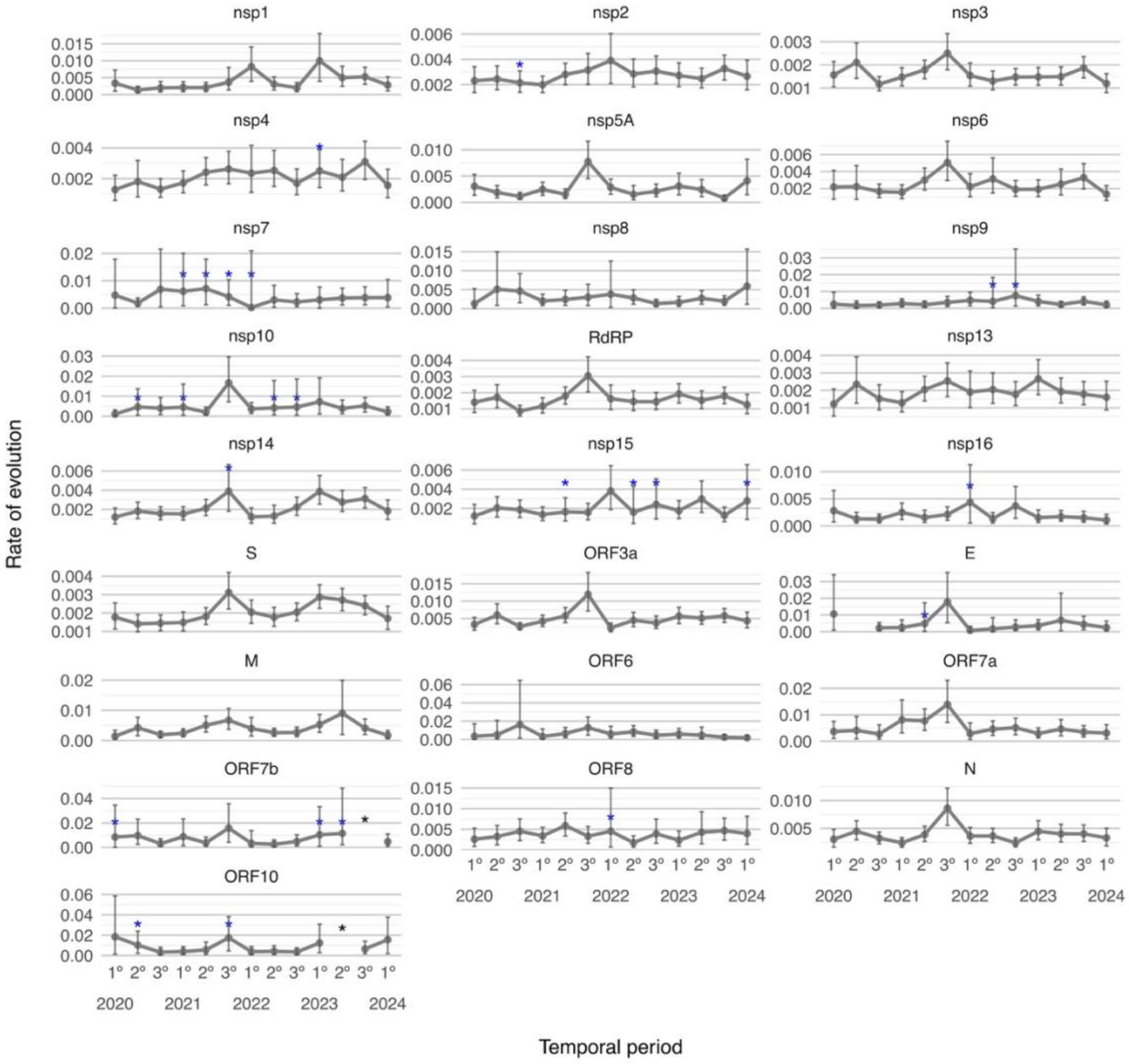

Figure S9. Selective pressure detected in average from the coding regions in each variant of concern and combination of variants of concern. The estimated nonsynonymous synonymous substitution rate ratio (*dN/dS*) for each of the 25 coding regions is presented in average, for each variant of concern, and for a combination of variants of concern (Mixture). Error bars indicate the 95% confidence interval provided by the estimation method, calculated as a mean of the 95% confidence intervals from the 25 coding regions, weighted according to the length of the corresponding coding regions. Estimates for each coding region at each VOC are shown in Figure 4.

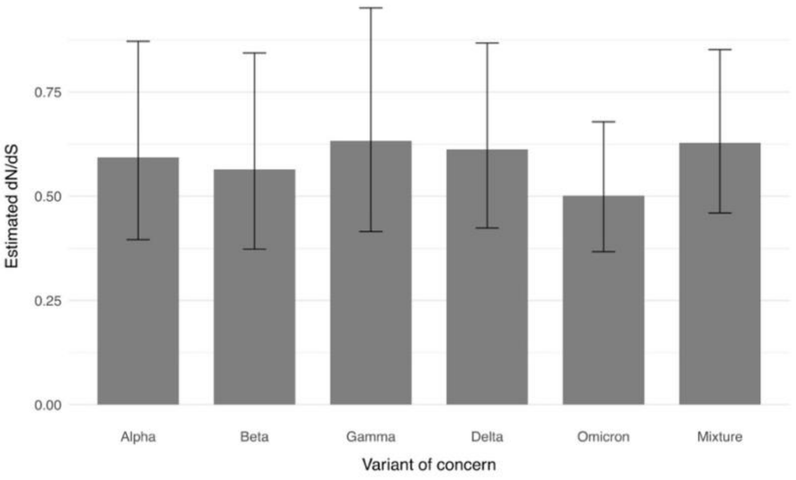

Figure S10. Number of synonymous and nonsynonymous codon changes observed in the coding regions at each variant of concern and combination of variants of concern. The total number of synonymous (blue) and nonsynonymous (red) codon changes observed across the 25 coding regions for each variant of concern, as well as for a combination of variants of concern (Mixture). The numbers of synonymous and nonsynonymous codon changes observed in each coding region for each VOC are shown in Figures S11 and S12.

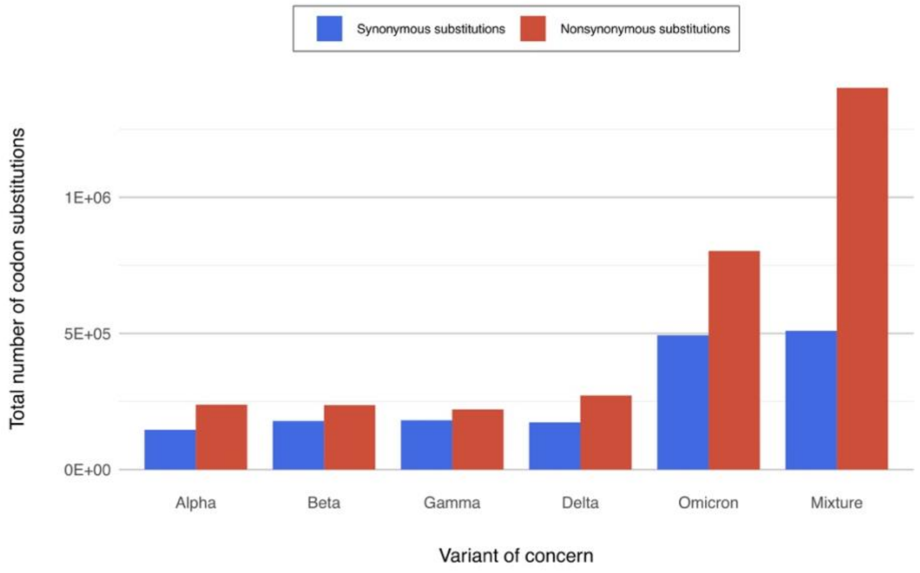

Figure S11. Number of synonymous and nonsynonymous codon changes observed in each coding region of each variant of concern and combination of variants of concern. For each coding region, the bars represent the number of observed synonymous (blue) and nonsynonymous (red) codon changes, for each variant of concern as well as for a combination of variants of concern (Mixture).

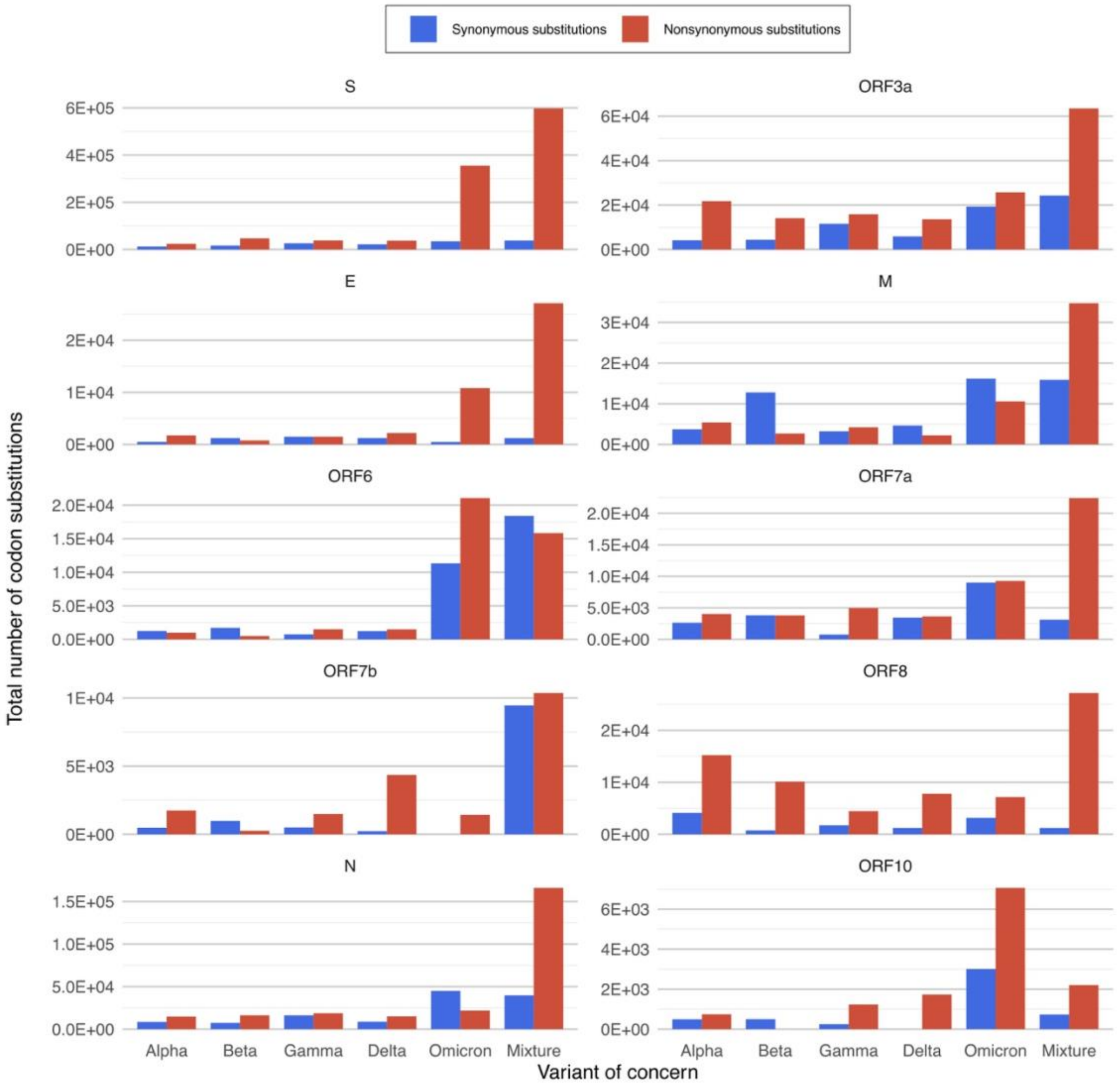

Figure S12. Number of synonymous and nonsynonymous codon changes observed in each non-structural protein coding region of ORF1ab at each variant of concern and combination of variants of concern. For each non-structural protein coding region ORF1ab, the bars represent the number of observed synonymous (blue) and nonsynonymous (red) codon changes, for each variant of concern as well as for a combination of variants of concern (Mixture).

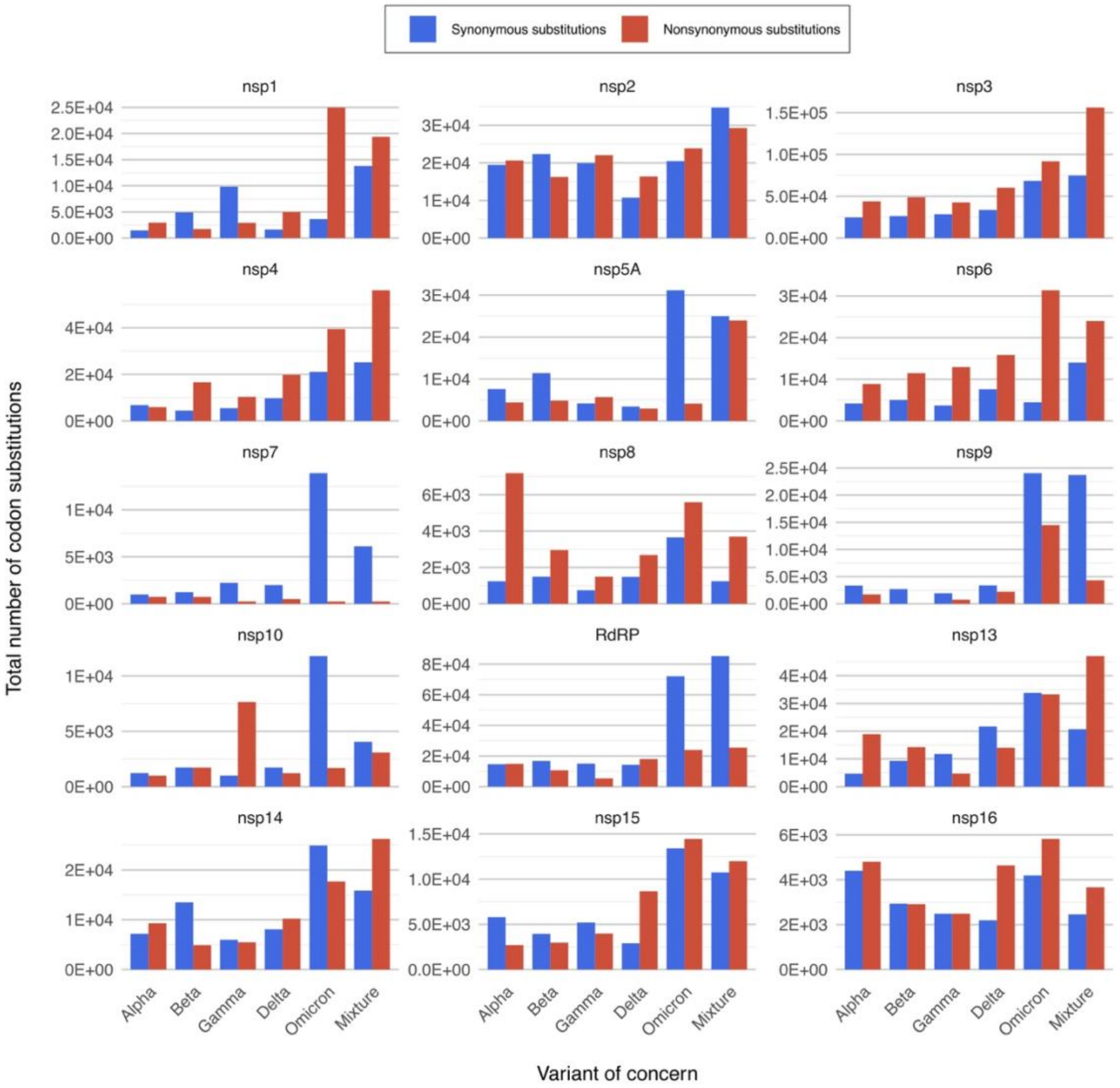

Figure S13. Selective pressure detected on average from the coding regions in each studied temporal period. The estimated nonsynonymous to synonymous substitution rate ratio (*dN/dS*) for each of the 25 coding regions is presented as an average for each studied temporal period. Error bars represent the 95% confidence interval provided by the estimation method, averaged across the 25 coding regions and weighted according to their respective lengths. Estimates for each coding region at each temporal period are shown in Figure S16.

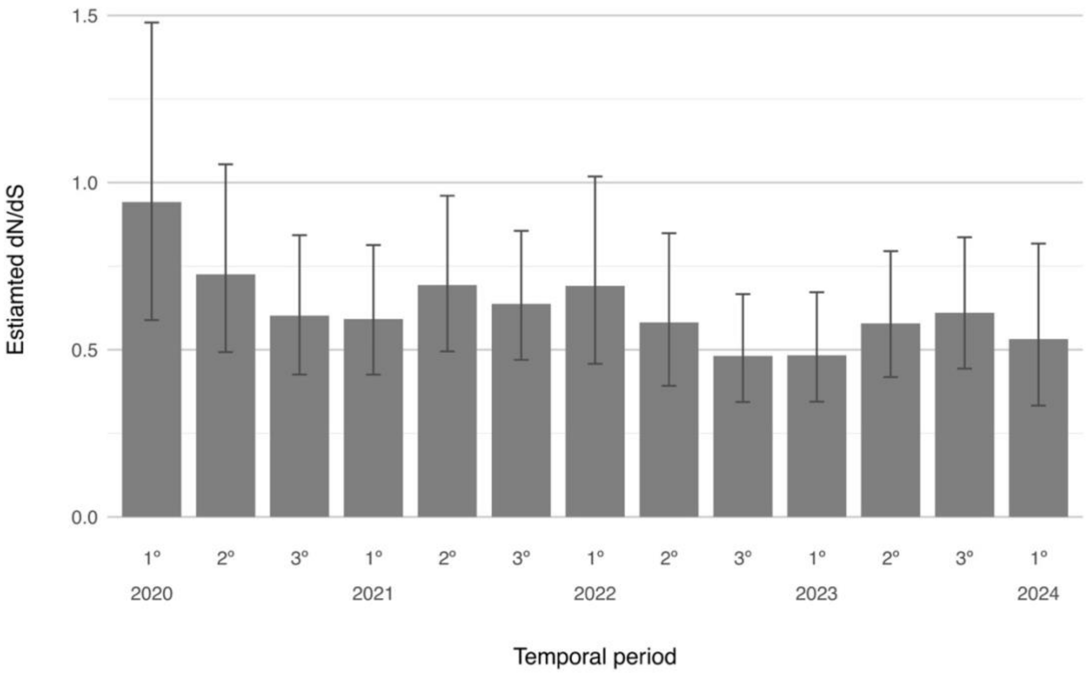

Figure S14. Number of synonymous and nonsynonymous codon changes observed in the coding regions at each studied temporal period. The observed number of synonymous (blue) and nonsynonymous (red) codon changes in the coding regions for each studied temporal period. Results for each coding region at each temporal period are shown in Figure S18.

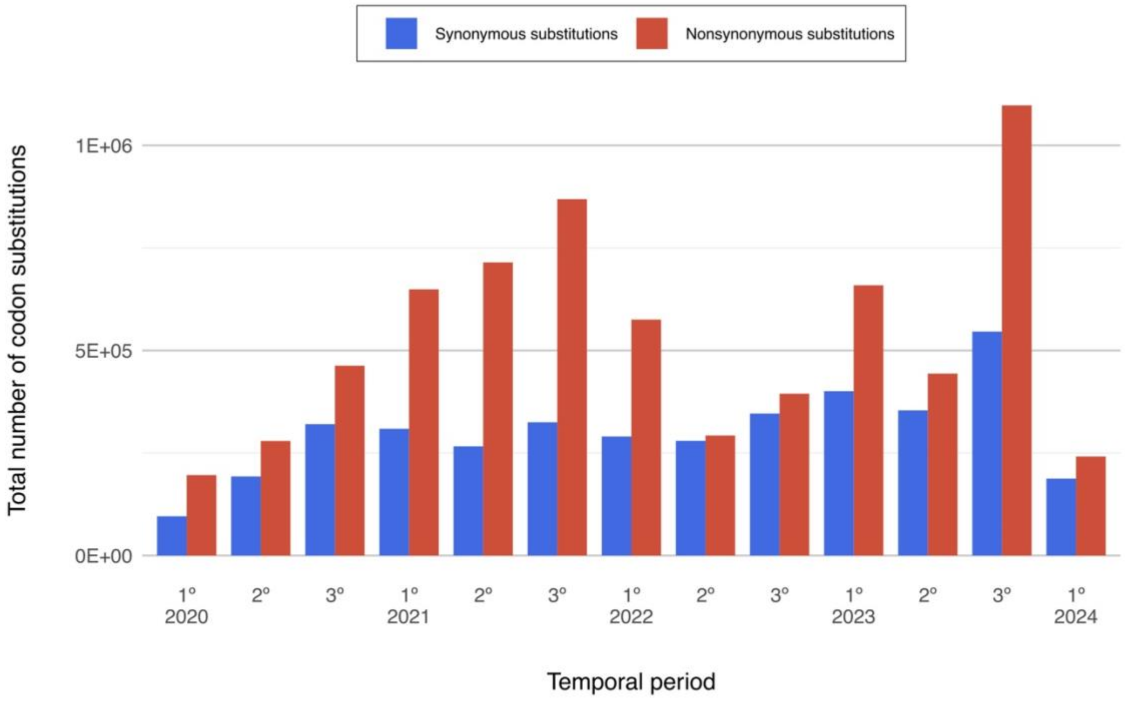

Figure S15. Selective pressure detected in each coding region considering all the studied temporal periods. The estimated nonsynonymous to synonymous substitution rate ratio (*dN/dS*) for each of the 25 coding regions, presented as an average considering all the studied temporal periods. Error bars represent the 95% confidence interval provided by the estimation method, as a mean of the 95% confidence intervals from the estimates at each temporal period. Estimates for each coding region at each temporal period are shown in Figure S16.

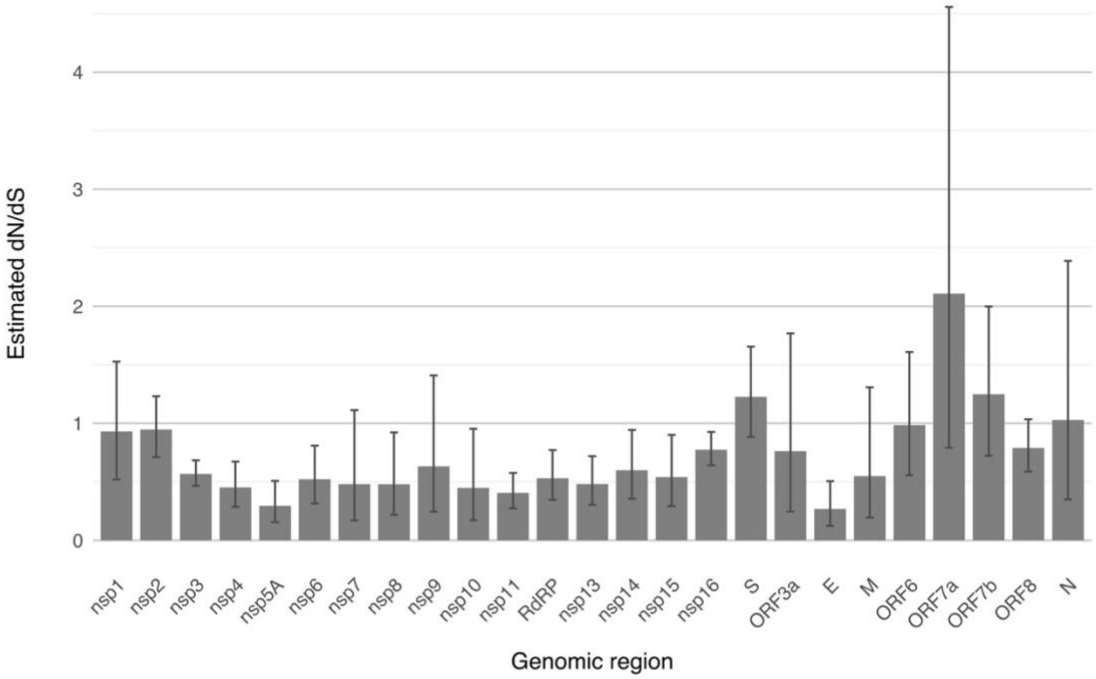

Figure S16. Selective pressure detected in each coding region at each studied temporal period. The estimated nonsynonymous to synonymous substitution rate ratio (*dN/dS*) for each of the 25 coding regions at each studied temporal period. Error bars represent the 95% confidence interval provided by the estimation method. Not available estimates (i.e. due to insufficient observed codon changes) are marked with asterisks.

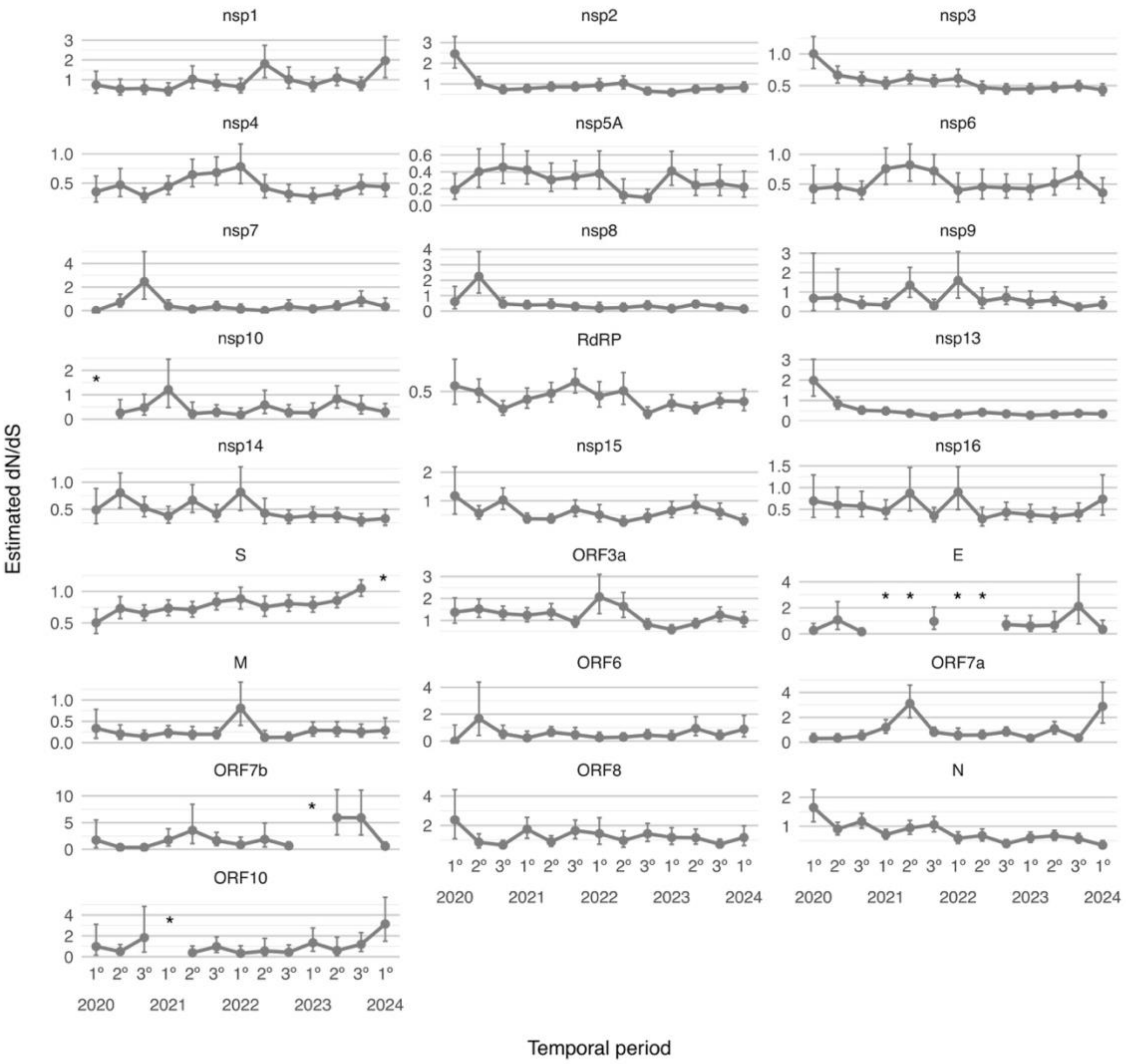

Figure S17. Number of synonymous and nonsynonymous codon changes observed in each coding region considering all the studied temporal periods. The observed number of synonymous (blue) and nonsynonymous (red) codon changes for each of the 25 coding regions, considering all the data from the studied temporal periods. Results for each coding region at each temporal period are shown in Figure S18.

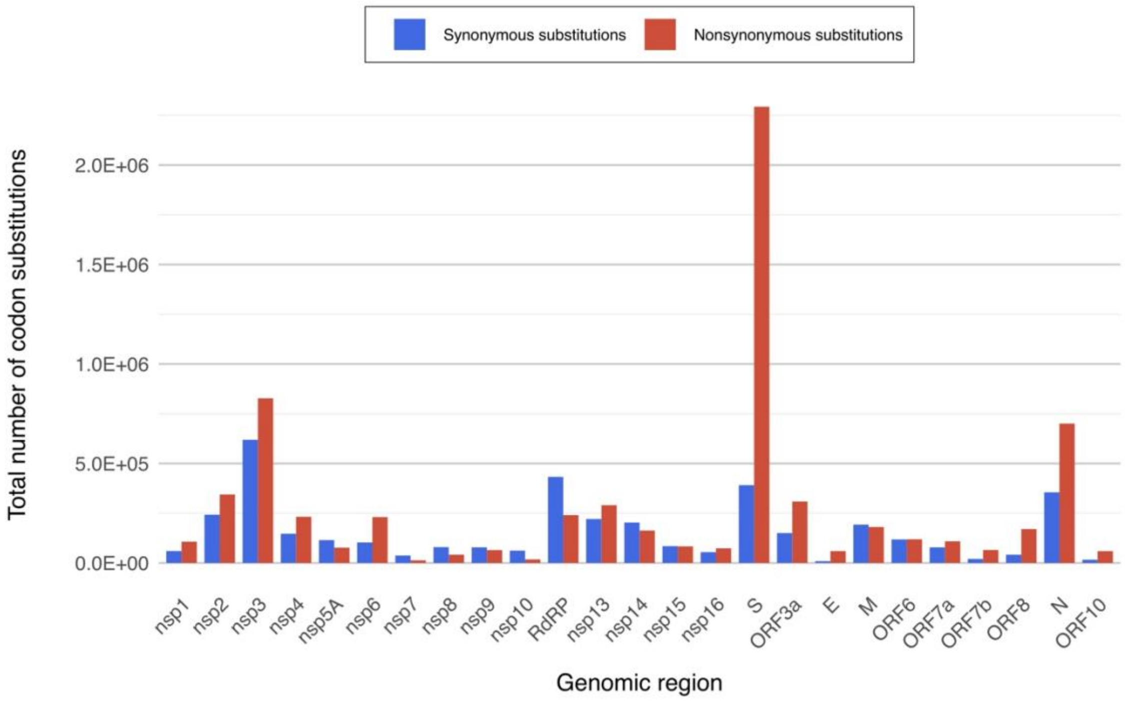

Figure S18. Number of synonymous and nonsynonymous codon changes observed in each studied temporal period. The observed number of synonymous (blue) and nonsynonymous (red) codon changes for each of the 25 coding regions across each studied temporal period.

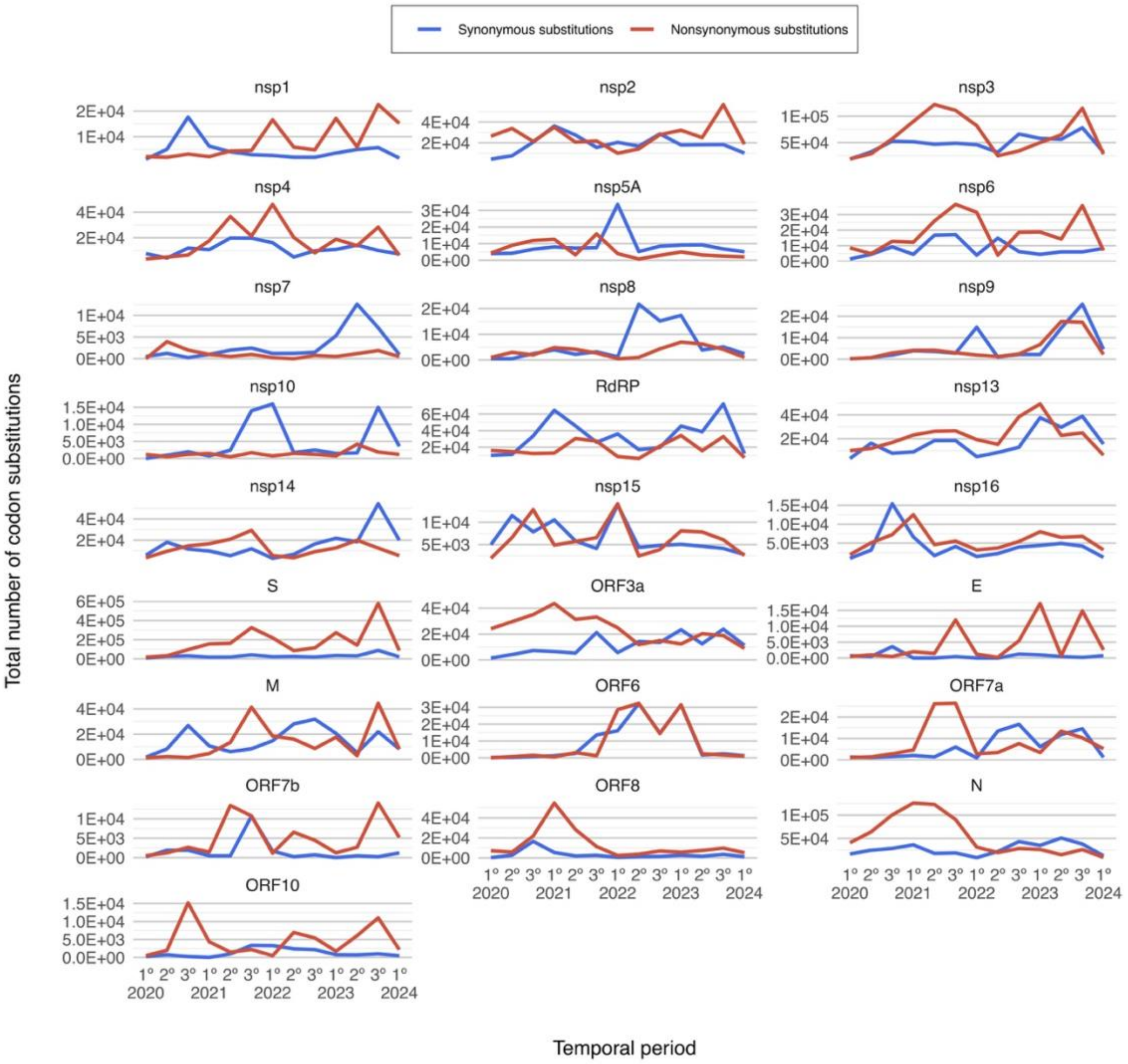

Figure S19. Distribution of codon site-specific selective pressure along the SARS-CoV-2 spike gene. Vertical bars indicate the number of independent datasets in which selection was detected at each codon site. Red and blue bars correspond to negatively selected sites (NSS) and positively selected sites (PSS), respectively. The representation below the plot shows the major structural domains of the spike protein – S1, S2, N-terminal domain (NTD), receptor-binding domain (RBD) with its receptor-binding motif (RBM), fusion peptide (FP), internal fusion peptide (IFP), heptad repeats 1 and 2 (HR1, HR2), transmembrane segment (TM) and cytoplasmic tail (CT) – facilitating the visual inspection of the detected selection signatures at the different functional regions of the protein.

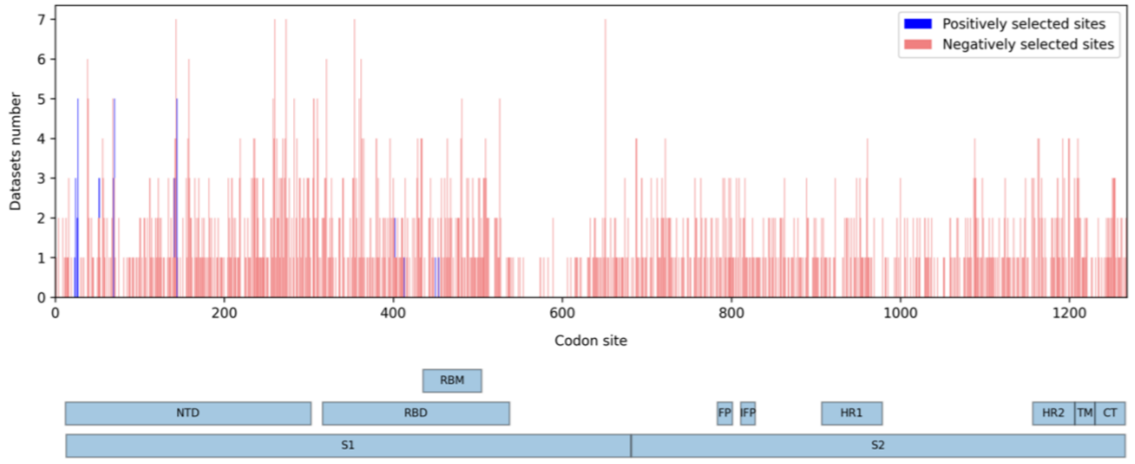

Figure S20. Significant positively selected sites identified in the SARS-CoV-2 Spike protein across different variants of concern. The significant positively selected sites are highlighted on the protein structure, with colors corresponding to each respective variant of concern. The protein structure (PDB code 6VXX) is used as an illustrative representation and it is shown from two visual perspectives. Notably, a higher number of PSSs are observed in the Omicron variant compared to earlier VOCs.

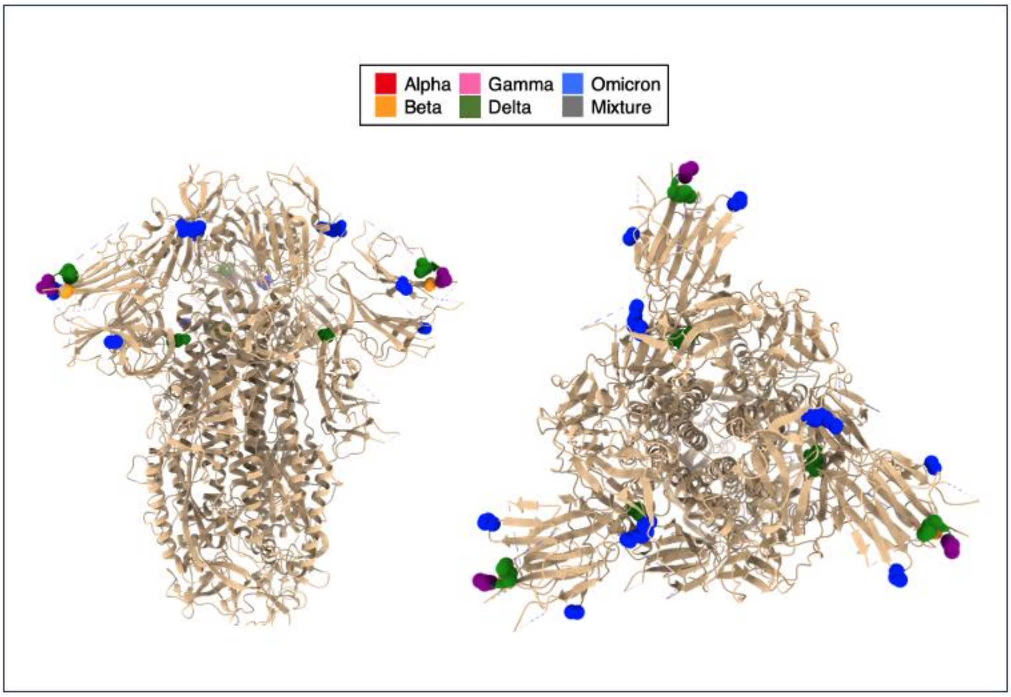

Supplement: Supplementary file 1 — Figure S1: Phylogenetic tree derived from the dataset with genome sequences from different VOCs. Figure S2: Nucleotide diversity at each studied temporal period for the SARS‐CoV‐2 coding regions (average). Figure S3: Nucleotide diversity for each SARS‐CoV‐2 coding region across all studied temporal periods (average). Figure S4: Nucleotide diversity for each SARS‐CoV‐2 coding region at each studied temporal period. Figure S5: Average rate of molecular evolution for coding regions in each variant of concern and combination of variants of concern. Figure S6: Average rate of molecular evolution for coding regions across each studied temporal period. Figure S7: Rate of molecular evolution in each coding region considering all studied temporal periods. Figure S8: Rate of molecular evolution of each coding region at each studied temporal period. Figure S9: Selective pressure detected in average from the coding regions in each variant of concern and combination of variants of concern. Figure S10: Number of synonymous and nonsynonymous codon changes observed in the coding regions at each variant of concern and combination of variants of concern. Figure S11: Number of synonymous and nonsynonymous codon changes observed in each coding region of each variant of concern and combination of variants of concern. Figure S12: Number of synonymous and nonsynonymous codon changes observed in each non‐structural protein coding region of ORF1ab at each variant of concern and combination of variants of concern. Figure S13: Selective pressure detected on average from the coding regions in each studied temporal period. Figure S14: Number of synonymous and nonsynonymous codon changes observed in the coding regions at each studied temporal period. Figure S15: Selective pressure detected in each coding region considering all the studied temporal periods. Figure S16: Selective pressure detected in each coding region at each studied temporal period. Figure S17: Number of synonymous and nonsynony [file JMV-97-e70604-s002.pdf]
